# Polyvinyl Alcohol-Based Membranes: A Review of Research Progress on Design and Predictive Modeling of Properties for Targeted Application

**DOI:** 10.3390/polym17081016

**Published:** 2025-04-09

**Authors:** Anca Filimon, Adina Maria Dobos, Mihaela Dorina Onofrei, Diana Serbezeanu

**Affiliations:** “Petru Poni” Institute of Macromolecular Chemistry, 41A, Grigore Ghica Voda Alley, 700487 Iasi, Romania; necula.adina@icmpp.ro (A.M.D.); mihaela.onofrei@icmpp.ro (M.D.O.); diana.serbezeanu@icmpp.ro (D.S.)

**Keywords:** polyvinyl alcohol, thermodynamic modeling, manufacturing membranes, environmental applications, biomedical fields

## Abstract

This review provides a comprehensive evaluation of the current state of polyvinyl alcohol (PVA)-based membranes, emphasizing their significance in membrane technology for various applications. The analysis encompasses both experimental and theoretical research articles, with a focus on recent decades, aiming to elucidate the potential and limitations of different fabrication approaches, structure–property relationships, and their applicability in the real world. The review begins by examining the advanced polymeric materials and strategies employed in the design and processing of membranes with tailored properties. Fundamental principles of membrane processes are introduced, with a focus on general modeling approaches for describing the fluid transport through membranes. A key aspect of discussion is the distinction between the membrane performance and process performance. Additionally, an in-depth analysis of PVA membranes in various applications is presented, particularly in environmental fields (e.g., fuel cell, water treatment, air purification, and food packaging) and biomedical domains (e.g., drug delivery systems, wound healing, tissue engineering and regenerative medicine, hemodialysis and artificial organs, and ophthalmic and periodontal treatment). Special attention is given to the relationship between membranes’ characteristics, such as material composition, structure, and processing parameters, and their overall performance, in terms of permeability, selectivity, and stability. Despite their promising properties, enhanced through innovative fabrication methods that expand their applicability, challenges remain in optimizing long-term stability, improving fouling resistance, and increasing process scalability. Therefore, further research is needed to develop novel modifications and composite structures that overcome these limitations and enhance the practical implementation of PVA-based membranes. By offering a systematic overview, this review aims to advance the understanding of PVA membrane fabrication, properties, and functionality, providing valuable insights for continued development and optimization in membrane technology.

## 1. Introduction

Membrane science and technology are widely recognized as powerful tools for addressing critical global challenges and driving the development of innovative industrial processes essential for sustainable growth [1]. As an advanced alternative to conventional separation methods, membrane technology is considered a key enabler of green chemistry and sustainable development. Its widespread adoption across diverse separation and purification applications is attributed to its high separation efficiency, low energy requirements, economic and environmental benefits, reduced investment costs, and potential for continuous operation [2,3]. However, the rapid advancement of membrane technology necessitates a fundamental understanding of its underlying mechanisms. A crucial aspect of optimizing membrane processes is the evaluation of mass transfer driven by external forces, which is essential for designing systems that operate under optimal conditions. In this context, modern membrane engineering plays a vital role in process intensification, employing innovative design strategies and process development methods to minimize the existing disadvantages of membrane technology, e.g., production costs, energy consumption, waste generation, etc. [4].

A membrane is a thin layer that acts as a selective barrier, effectively separating two distinct phases, which may be either liquid or gas. In other words, in separation processes, membranes permit the passage of a specific component from a mixture while restricting the movement of others. The study of membranes dates back to the 18th century, with Abbe Nolet introducing the term “osmosis” in 1748 to describe the movement of water through a diaphragm [5]. A fundamental property of membranes is their ability to control the transport of chemical species, enabling selective separation. To perform this function effectively, membranes must possess specific structural characteristics. They are generally classified as either porous or non-porous. Porous membranes contain well-defined pores that facilitate separation based on size exclusion, whereas non-porous membranes rely on the chemical properties of the polymer matrix to regulate molecular diffusion, favoring the passage of particular species. Membrane performance is typically governed by a balance between permeability—the normalized flux of the target component—and selectivity, which is defined as the concentration ratio of the desired species in the permeate stream. Achieving both high permeability and high selectivity simultaneously remains a significant challenge. The structural configuration of a membrane plays a key role in determining these properties: dense membranes exhibit high selectivity but lower permeability due to their thickness, whereas porous membranes allow higher fluxes but with reduced selectivity. An optimal membrane design often features an asymmetric structure, consisting of a highly porous support layer that enhances permeability and mechanical stability, combined with an ultrathin dense selective layer that ensures effective separation. While asymmetric membranes offer numerous advantages, challenges persist. These include precisely controlling their structural properties and scaling up production for industrial applications [6].

With the growing global population, expansion of industries, numerous droughts, and lack of proper management in water consumption, water scarcity and energy consumption are becoming critical challenges [7,8]. Membrane technology, driven by a multidisciplinary approach, has emerged as a promising solution, finding applications across various industries, including water treatment, chemical processing, pharmaceutical, biotechnology, energy, and food [9,10]. Advances in membrane technology have expanded its role in processes, such as pervaporation (PV), ultrafiltration (UF), microfiltration (MF), reverse osmosis (RO), and electrodialysis, while also enabling innovations in forward osmosis (FO), membrane reactors (MR), fuel cells (FC), dehumidification, electrochemical energy storage (EES), and lithium-ion batteries [11,12,13].

In the given context, membrane technology has covered widespread industrial and environmental applications due to its numerous advantages as a clean and energy-efficient separation process. Compared to conventional methods such as filtration, distillation, ion exchange, and chemical treatment, membrane separation offers a more sustainable alternative by reducing energy consumption and minimizing the need for chemical additives. One of its key benefits is the ability to perform continuous separation under mild operating conditions, making it highly suitable for various industrial processes. Additionally, membrane technology provides flexibility in system design and can be integrated with other separation techniques to create hybrid processes, enhancing overall efficiency and selectivity. It also enables the production of high-quality products, making it particularly valuable in industries such as water purification, food processing, pharmaceuticals, and chemical manufacturing [6].

Despite its advantages, membrane technology faces several challenges that limit its broader implementation. Major obstacles include membrane fouling, which can reduce separation efficiency and increase maintenance costs. Other drawbacks involve a relatively low membrane lifetime, limited selectivity, and reduced flux over time. However, these issues are intrinsic to the separation process and can be mitigated by advances in membrane materials’ development, surface modifications, and process optimization [6]. Considering the stated facts, a key factor enabling the expansion of membrane applications into more demanding environments is the continuous development of high-performance membrane materials. In this way, advances in material science have led to the design of membranes with improved thermal, chemical, and mechanical stability, allowing them to operate under extreme conditions while maintaining efficiency and durability. To further enhance their performance, customized membranes can be developed by carefully selecting suitable materials, optimizing fabrication techniques, and refining membrane configurations.

It is well known that the membrane material is the key element of membrane processes, directly influencing both process efficiency and practical applicability. Moreover, different application fields impose specific requirements on membrane materials to meet their unique operational demands [14,15]. A wide range of polymers has been utilized for fabricating membranes using various techniques, such as phase inversion, electrospinning, sintering, melt-spinning, cold-stretching, and 3D printing [8]. For example, typically, in fuel cell applications, cationic or anionic functionalized polymers have been developed for membrane fabrication. Among the various polymer classes, perfluorosulfonic acid-based copolymers, sulfonated hydrocarbon and aromatic polymers, acid-doped polybenzimidazole (PBI), polyphosphazene, and various quaternized polymers have been extensively studied, ranging from laboratory research to pilot-scale implementation [9,16,17,18]. These materials are selected for their ability to facilitate ion transport while maintaining mechanical and chemical stability under fuel cell operating conditions. In particular, polymer electrolyte membranes (PEMs) play a crucial role in fuel cells, where their functional properties include optimal water absorption, low electrical resistance, high proton/hydroxide ion conductivity, minimal methanol permeability, excellent gas barrier properties, and superior mechanical strength. It can be stated that these attributes make PEMs a preferred choice for fuel cell applications, ensuring efficient energy conversion and long-term performance [19,20]. Following the same research direction, for water treatment applications, including RO, NF, and FO processes, various polymeric materials have also been utilized for fabricating thin selective layers. These include cellulose-based polymers, crosslinked aromatic polyamides, sulfonated polymers, crosslinked semi-aromatic and aliphatic polyamides, polyelectrolytes, water-soluble polymers, and biopolymers [9,21]. Each material is selected for its ability to provide high separation efficiency, chemical resistance, and durability in different water treatment environments.

Starting from these findings, the choice of polymer depends on the intended application, being guided by certain desired characteristics, including solubility in environmentally friendly solvents, high mechanical and thermal stability, chemical resistance, and efficient separation capabilities [22,23,24]. Additionally, identifying suitable materials requires further investigation into controlling the nature and magnitude of interactions between permeants and membranes, as well as the packing density and segment mobility of polymer chains. While both material selection and membrane fabrication techniques influence the transport mechanisms, stability, and overall performance, the fabrication process plays a crucial role in defining membrane morphology, which in turn affects permeation rates through its physical properties. However, as membrane technology advances and environmental conditions become increasingly complex, the demand for high-performance membranes continues to rise—in particular, the development of membranes capable of operating in demanding environments. To meet these demands, researchers are exploring advanced polymeric materials with enhanced stability, selectivity, and resistance to challenging conditions [25,26].

In this context, poly(vinyl alcohol) (PVA) is a synthetic, non-toxic polymer that combines the above-mentioned properties with an excellent film- and fiber-forming ability, good chemical and mechanical stability, and tuned hydrophilicity, as well as emulsifying and adhesive properties, making it an extremely versatile candidate in membrane technology. Since the 1970s, substantial progress has been made in both PVA membrane preparation methods and its diverse range of applications [27,28,29,30].

Unlike conventional vinyl polymers, PVA is not directly synthesized from its monomer, vinyl alcohol, due to the latter’s instability and spontaneous conversion into acetaldehyde. Instead, it is obtained through the controlled hydrolysis of polyvinyl acetate (PVAc), where acetate (-COOCH_3_) groups are replaced by hydroxyl (-OH) groups in the presence of a catalyst [31,32]. Depending on the degree of hydrolysis (DH), PVA can exist in partially hydrolyzed or fully hydrolyzed forms. The degree of hydrolysis is crucial in determining PVA’s physicochemical properties, such as solubility, crystallinity, and mechanical behavior. Another key characteristic of PVA is its molecular weight, which typically ranges from 9000 to 186,000 g/mol and influences viscosity, film strength, and fiber formation. Additionally, the degree of hydrolysis (partial or complete) affects the balance between water solubility and structural integrity. Fully hydrolyzed PVA exhibits high crystallinity and limited water solubility due to extensive intermolecular hydrogen bonding, making it suitable for applications requiring durability and chemical resistance. Partially hydrolyzed PVA, in contrast, retains some acetate groups, resulting in improved processability and enhanced water solubility, which is advantageous for applications requiring controlled swelling and permeability.

**Figure 1:**
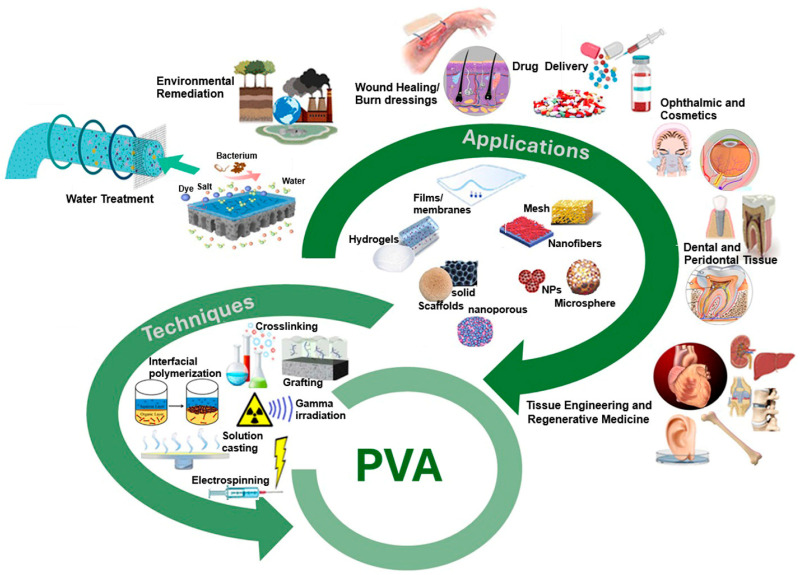
Schematic representation of the PVA-based membrane applications.

On the other hand, the presence of hydroxyl groups in PVA’s backbone confers strong hydrophilicity, promoting interactions with water molecules and enhancing its affinity for aqueous environments. This property is particularly beneficial in membrane applications, where selective permeability, moisture retention, and antifouling characteristics are essential. Beyond its hydrophilic nature, PVA also possesses biocompatibility, biodegradability, non-toxicity, high optical clarity, thermal stability, and robust mechanical strength, making it a highly adaptable material for diverse applications (Figure 1). Thus, as a result of these characteristics, PVA is an attractive polymer for the fabrication of hydrogels, thin films, scaffolds, electrospun fibers, composites, and especially polymeric membranes [33,34]. Therefore, due to this unique combination of properties, PVA has found extensive use in membrane technology, both as a base polymer and as a functional additive to improve hydrophilicity, mechanical durability, and separation efficiency. It is widely employed in water purification, gas separation, pervaporation, and biomedical applications, where controlled transport properties are essential [34,35]. Moreover, its ability to form fibers and films makes it ideal for electrospun nanofiber membranes, hollow fiber membranes, and flat-sheet configurations. Fibrous membranes, in particular, offer high surface area and interconnected porosity, which enhance permeability and selectivity, making them suitable for filtration, tissue engineering, and protective coatings.

Despite its excellent properties, PVA also exhibits certain limitations, including ion permeability, a high degree of swelling, compaction under pressure, and reduced flux when highly crosslinked. Additionally, it approaches its melting and degradation temperature relatively quickly. In recent years, extensive research has been conducted to address these drawbacks [36,37]. These studies have contributed to expanding PVA’s potential applications by providing valuable insights into its stability and performance under various conditions.

With continuous advances in polymer engineering, PVA-based membranes have been extensively studied between 2000 and 2025, owing to their remarkable adaptability. These membranes are of particular interest not only due to their potential for functional diversification and derivatization through innovative modification strategies, but also for their expanding applicability in the biomedical, environmental, and industrial domains. Their unique combination of hydrophilicity, biocompatibility, chemical stability, and mechanical robustness makes them indispensable in high-performance membrane technologies. In the following sections, based on the synthesis, comparison, and careful evaluation of the relevant literature from the Web of Science database, the versatility, tunability, and durability of PVA-based membranes are presented, positioning them as key materials in advanced membrane applications. Furthermore, the emerging trends, current challenges, and future perspectives are discussed, particularly in the context of improving selectivity, permeability, stability, and process scalability. By addressing the latest research developments and identifying gaps in the field, this review aims to provide a deeper understanding of PVA membrane technologies and guide future innovations in their design and application.

## 2. Thermodynamic Modeling of Polymer Solutions: Polymer System Prediction and Membrane Performance Modeling

Thermodynamic modeling of polymeric systems has gained significant attention, particularly in the study of phase equilibrium, as it plays a crucial role in various industrial applications. A deep understanding of the phase behavior of polymer solutions is essential for accurately predicting suitable polymer systems and optimizing membrane performance. Given that many polymeric materials are synthesized from solution, phase behavior analysis is fundamental for designing membranes with tailored properties for specific applications. Thus, an accurately predicted polymer system not only enables the precise design of membrane performance through optimal material selection but also aids in the development and optimization of manufacturing processes and the modeling of separation mechanisms across different operational modes—key aspects of membrane technology. Consequently, selecting the appropriate polymer system and employing robust modeling techniques are indispensable tools in membrane research and industry. These approaches enhance the understanding of separation mechanisms in filtration, offering significant opportunities to improve productivity and reduce costs.

Based on this hypothesis, miscible polymer blends are of great practical significance, as they enable the development of a wide range of functional materials by combining existing polymers. For instance, polymer blend membranes are only effective when they integrate the properties of two or more polymers simultaneously [38,39]. Blending also facilitates material processing by modifying flow properties, while the diffusion behavior of individual species, which affects the kinetics of mixing and phase separation, can be controlled by understanding how blending influences their mobilities. Moreover, the study of miscible polymer blends provides valuable insights into the role of intrinsic dynamic properties and intermolecular interactions in governing polymer dynamics [38]. By varying the composition of miscible blends, it is possible to investigate how interchain dynamic constraints modify chain dynamics while maintaining a fixed intramolecular structure. A key objective in this context is to tailor blend properties based on the known characteristics of their constituent polymers. However, it is important to acknowledge that polymer blends are not always fully compatible, as most exhibit only partial miscibility [40]. According to thermodynamic principles, a polymer mixture is considered stable when the Gibbs free energy of mixing (ΔG_M_, Equation (1)) has a negative value, ΔG_M_ < 0, and its second derivative with respect to the volume fraction of species is positive, $\left(\frac{\partial^{2}\Delta G_{M}}{\partial \phi_{i}^{2}}\right)>0.$ These conditions ensure both the thermodynamic favorability of mixing and the absence of phase separation, leading to a homogeneous and stable polymer blend [41].

(1) $$\frac{{\Delta G}_{M}}{RT}=\left(x_{1}\ln\phi_{1}+x_{2}ln\phi_{2}\right)+gx_{1}\phi_{2}=\frac{{\Delta G}_{M}^{C}}{RT}+\frac{{\Delta G}_{M}^{R}}{RT},$$

where the parameters involved are the gas constant—R, temperature (K)—T, mole fractions of components—x_i_, volume fractions of components—$\phi_{i}$, and polymer-solvent interaction parameter—g. The sum $\left(x_{1}ln\phi_{1}+x_{2}ln\phi_{2}\right)=\frac{{\Delta G}_{M}^{C}}{RT}$, of purely statistical origin, is called the combinatorial part, whereas the non-combinatorial part, $gx_{1}\phi_{2}=\frac{{\Delta G}_{M}}{RT}$, constitutes the so-called reduced residual Gibbs energy of mixing.

The Gibbs free energy of a polymer mixture is a key parameter in predicting the phase behavior of polymeric systems and can be derived from various thermodynamic theories. One of the earliest and most widely used models is the statistical thermodynamics theory proposed by Flory and Huggins [42,43]. Over time, additional models have been developed to address the limitations of pre-existing theories, such as the compressible regular solution model (CRSM) [44]. Selecting an appropriate thermodynamic model for a given polymer blend system enhances the accuracy of miscibility and compatibility predictions, ultimately facilitating the successful fabrication of polymer membranes [44,45].

**Figure 2:**
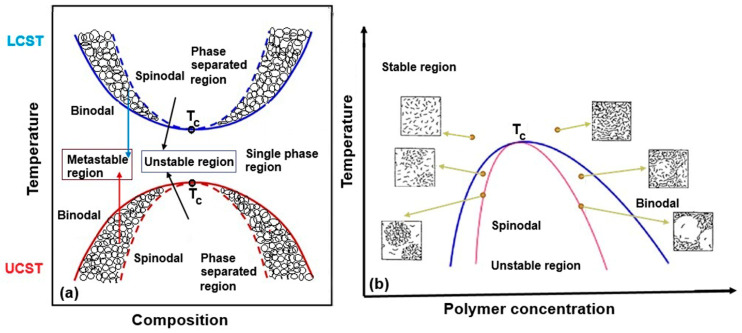
(**a**) Phase diagram: temperature versus composition for a binary polymer blend (LCST—lower critical solution temperature; UCST—upper critical solution temperature). (**b**) Schematic representation of phase-separation mechanisms in polymer solutions: phase separation occurs via the formation of small, isolated spherical regions of the second phase, which grow over time (nucleation and growth); phase separation leads to interconnected, worm-like structures that evolve into coarsened spheroidal domains (spinodal decomposition). Adapted with permission from [44].

Studying the phase diagram is crucial before initiating the preparation or processing of membranes, as it provides insights into the behavior of polymer mixtures under varying conditions. In the case of miscible polymer mixtures subjected to changes in temperature or solvent removal (via evaporation or non-solvent addition), two distinct phase-separation mechanisms can occur when the phase diagram boundary is crossed: nucleation and growth, and spinodal decomposition, which leads to interconnected phase domains (Figure 2). These mechanisms depend on the polymer concentration and thermodynamic conditions, influencing the final morphology of polymer-based materials. While spinodal decomposition has largely been of academic interest in the study of polymer blends, its practical significance has grown in the context of membrane formation, particularly for solvent–polymer phase separation via non-solvent addition [44].

**Figure 3:**
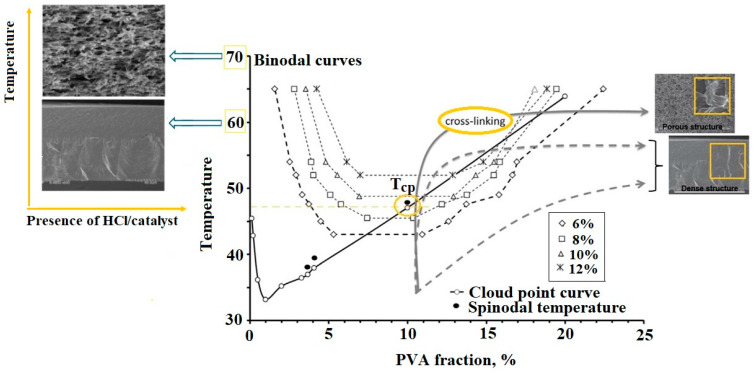
Phase diagram of PVA/water solution showing the pathways leading to the formation of dense and porous membranes as a result of the crosslinking reaction in the diphasic region and the effect of temperature and catalyst on the membrane structure. Adapted with permission from [47].

This aspect was emphasized by Razmgar et al. [46] who experimentally validated the phase diagram prediction in the preparation of membranes using a poly(vinylidene fluoride) (PVDF)/polyvinyl alcohol (PVA) blend. The study showed that the miscibility of the binary system, determined by the enthalpy of mixing, indicated partial miscibility, with compatibility occurring only at very low (0.01–0.03) or very high (0.91–1.00) PVDF weight fractions. As a result, the miscibility range for obtaining compatible blends and membranes corresponded to a mixing enthalpy between 0.001 and 0.01 cal/mol. Similarly, M’Barki et al. [47] prepared porous membranes through temperature-induced phase separation of PVA with a degree of hydrolysis (DH) of 72%. In this case, the phase-separation mechanism was spinodal decomposition, and the authors studied the binary PVA/water phase diagram (Figure 3) and crosslinking kinetics to determine the optimal conditions for porous membrane preparation. This underscores the importance of phase diagram analysis in guiding the membrane fabrication process. Further modeling studies on the PVA/water system were realized by the same group [48], revealing that the presence of volatile catalytic components, such as hydrochloric acid (HCl), accelerates the reaction between glutaraldehyde and PVA, leading to rapid crosslinking. This reaction occurs simultaneously with phase inversion and affects the membrane formation process. Thus, since the phase-separation process is conducted under open conditions, mass transfer phenomena play a significant role in determining membrane morphology. In this case, both water and HCl evaporate during the process. This evaporation changes the composition of the solution, affecting diffusion and phase separation. Because HCl participates in mass transfer, it alters the rate at which the system moves from a monophasic to a diphasic state. Faster removal of small molecules like HCl and water can lead to different pore structures due to the changing diffusion gradients. In the diphasic region, where phase inversion leads to pore formation, the connectivity between emerging pores is influenced by the presence of HCl. Therefore, the numerical model developed for this study accounts for these mass transfers and demonstrates that solvent and catalyzer evaporation must be carefully controlled to achieve the desired porous structure. This underscores the complexity of membrane fabrication and the need to consider various factors influencing phase separation. Thus, understanding the phase behavior of polymer mixtures through a phase diagram is essential for predicting phase-separation mechanisms and optimizing conditions for membrane preparation. Complementing this, Young and Chuang [49] conducted a thermodynamic analysis using Flory–Huggins’s ternary solution theory to examine membrane formation in PVA/water/DMSO mixtures. Their study provided valuable estimations of the optimal solution ratios that favor system demixing, further confirming the importance of understanding phase behavior in predicting and optimizing membrane formation processes.

Following the same research direction, Reuvers et al. [50] developed a model to predict membrane formation conditions during immersion precipitation, specifically determining whether phase separation follows a liquid–liquid mechanism. This model integrates three components: a solvent, a non-solvent, and a polymer. During immersion precipitation, phase separation can occur through either instantaneous or delayed demixing. The authors also formulated equations to describe the boundary conditions at the interface between the polymer solution and the coagulation bath, focusing on isothermal diffusion and mass transfer.

Boom et al. [51] expanded further the theory of membrane formation by developing a model for a quaternary system comprising two miscible polymers, a solvent, and a non-solvent, namely poly(ether sulfone)/poly(vinylpyrrolidone)/N-methylpyrrolidone/water. This model highlights the effects of polymeric additives (e.g., poly(vinylpyrrolidone), PVP) and their role in the formation process of membranes (to fine-tune membrane structure and performance). Thus, the presence of PVP in the polymer solution accelerates the onset of phase separation. Transmission experiments have shown that demixing starts earlier when PVP is added, suggesting that the additive reduces the energy barrier for phase separation. Although demixing is accelerated initially, PVP regulates the diffusion rate of water (non-solvent) and solvent molecules, leading to a more uniform pore structure. This contrasts with the behavior of ternary systems without additives, where demixing can be delayed depending on the solvent–nonsolvent exchange kinetics.

These studies demonstrate that phase diagram analysis, coupled with thermodynamic modeling, plays a pivotal role in understanding the mechanisms of phase separation and guiding the preparation of membranes with desired properties to specific technological applications. Furthermore, by synthesizing and analyzing the existing research, the trend of progress in thermodynamic modeling and membrane performance prediction was observed, emphasizing the integration of computational techniques and empirical models to improve accuracy and efficiency. Thus, the advances in thermodynamic modeling of polymer solutions with impact on membrane performance have introduced several innovative approaches, namely (i) combining classical thermodynamic theories (e.g., Flory–Huggins, UNIFAC) with experimental validation to improve accuracy [52,53]; (ii) the integration of machine-learning algorithms and molecular simulations to refine thermodynamic predictions [54,55]; (iii) modeling alternative solvent systems that align with sustainability/environmental friendly goals in membrane production [55,56].

Beyond material selection and processing, membrane efficiency also depends on several factors, including high water flux, solute rejection performance, module configuration, and mechanical, chemical, and thermal stability, as well as large-scale processability and cost-effective operating conditions. Once the processing parameters are optimized, the next critical step involves modeling and simulating filtration processes (e.g., ultrafiltration, microfiltration, and reverse osmosis) to predict membrane performance under real-world conditions. Filtration processes involve complex, time-dependent flow patterns with irregular fluctuations, which can be analyzed using hydrodynamic theories and empirical equations, such as Darcy’s Law, the Hagen–Poiseuille equation, and the Carman–Kozeny equation [57,58]. The choice of an appropriate model depends on the specific membrane structure, including the pore size, shape, porosity, average capillary length, pore-size distribution, surface area, and tortuosity, as these factors directly influence transport mechanisms (Table 1). For instance, in practical applications, ultrafiltration and microfiltration systems require different modeling approaches compared to reverse osmosis, where solute transport and pressure-driven flow behave differently.

Integrating the thermodynamic modeling of polymeric systems with hydrodynamic simulations of filtration processes provides a comprehensive framework for membrane design, predicting their performance, as well as optimizing the process. Thus, this approach ensures that membranes not only possess the desired structural characteristics but also operate efficiently in their intended applications.

The efficiency of a membrane is primarily dictated by the structure of its pores and the physicochemical properties of the material, which directly influence transport mechanisms and separation capabilities. Membranes are commonly classified based on their pore size, a criterion widely accepted in the literature [67,68]. This classification includes microfiltration (MF), ultrafiltration (UF), nanofiltration (NF), and reverse osmosis (RO) membranes. MF membranes, with pore sizes ranging from approximately 0.1 to 5 μm, are particularly effective in the removal of suspended solids, bacteria, and large macromolecules. UF membranes, characterized by pore sizes between 0.01 and 0.1 μm, facilitate the separation of proteins and colloidal particles, making them suitable for applications such as wastewater treatment and bioprocessing. NF membranes, with pore sizes of 1–10 nm, allow for the selective retention of divalent and larger monovalent ions, playing a crucial role in water softening and molecular separations within the food and pharmaceutical industries. RO membranes, possessing pore sizes below 1 nm, operate primarily through solution-diffusion mechanisms rather than size exclusion, making them indispensable for desalination and ultrapure water production.

Beyond pore size, membrane performance is influenced by additional structural and surface properties, including hydrophilicity, surface charge, and mechanical stability. Hydrophilic membranes demonstrate reduced fouling tendencies due to their ability to resist organic contaminants and protein adsorption, a characteristic extensively explored in water treatment and biomedical applications. Surface charge plays a critical role in NF and RO membranes, where electrostatic interactions govern ion rejection efficiency. Moreover, mechanical robustness is essential for membrane longevity, particularly in high-pressure applications, as evidenced in studies on both polymeric and ceramic membranes [9].

In conclusion, membranes’ performance is determined by a combination of pore structure, surface properties, and mechanical integrity, all of which influence their separation capabilities. The integration of thermodynamic and hydrodynamic modeling provides a fundamental framework for optimizing membrane design, enabling enhanced functionality and broadening their application potential across various scientific and industrial domains.

## 3. Methods for Manufacturing PVA-Based Membranes

A comprehensive review of the literature reveals significant advancements in the techniques used for developing polymeric membranes, both at the industrial and laboratory scales [69,70]. In particular, the fabrication of polyvinyl alcohol (PVA)-based membranes employs a diverse range of chemical and physical methods, each carefully tailored and controlled to enhance specific membrane properties. These methods play a crucial role in determining the performance, durability, and suitability of PVA-based membranes for various applications. Optimizing these techniques is essential for the development of advanced materials in the environmental, biomedical, and industrial fields.

A notable shift towards eco-friendly and sustainable membrane fabrication methods has been observed, in accordance with the principles of Green Chemistry [71,72]. Key strategies promoting sustainability include the following: (i) the utilization of green solvents, such as water-based systems, ionic liquids, or solvent-free approaches, to minimize hazardous waste generation; (ii) the implementation of eco-friendly fabrication techniques, favoring physical processes over chemical ones to reduce toxic byproducts; (iii) the incorporation of environmentally friendly additives, which not only diminish the reliance on hazardous chemicals but also improve the biodegradability of the membranes.

### 3.1. Crosslinking

Crosslinking is an important technique in the fabrication of PVA-based membranes, significantly enhancing their mechanical and chemical stability. This process can be achieved through various chemical and physical methods [73,74]. In chemical crosslinking, agents such as glutaraldehyde, boric acid, and citric acid react with functional groups in PVA, forming covalent or ionic bonds that reinforce the membrane structure. The reaction typically occurs in an acidic environment, with sulfuric and hydrochloric acids being commonly used. However, due to the potential toxicity of crosslinking agents, careful measures must be taken to eliminate residual chemicals from the final material. Beyond chemical approaches, physical crosslinking methods, such as freeze/thaw cycling, heat treatment, and gamma irradiation offer alternative strategies [75,76]. Radiation-induced crosslinking, for example, employs gamma rays or electron beams to create crosslinks without the need for chemical additives. This technique results in membranes with superior thermal and chemical resistance, improving water resistance and durability—critical attributes for applications requiring prolonged membrane stability. Such membranes are widely used in water purification, drug delivery, and biomedical applications, where preventing excessive swelling or dissolution in aqueous environments is essential.

The importance of crosslinking in membrane fabrication has been extensively studied. One of the earliest comprehensive reports, presented by Bolto et al. [73], explored various crosslinking processes for pristine PVA membranes, emphasizing their influence on swelling behavior in reverse osmosis applications. Building on this, Maiti et al. [77] conducted a detailed review of PVA-based nanocomposite membranes, particularly in the context of direct methanol fuel cells (DMFCs), focusing on structural modifications, transport properties, and performance improvements.

Following the same direction, Wu et al. [78] demonstrated the successful preparation of a composite PVA ultrafiltration membrane for oily water treatment. By crosslinking PVA to a mixed cellulose ester microfiltration membrane, the resulting material exhibited excellent antifouling properties against oil contamination. Similarly, Wang et al. [79] investigated the effects of chemical crosslinking with glutaraldehyde and sulfuric acid, as well as heat treatment, on porous PVA membranes. Their study revealed that while chemical crosslinking had minimal impact on membrane structure, heat treatment enhanced crystallinity, leading to morphological changes. The treated membranes were systematically evaluated for water permeation flux, morphology, and mechanical properties, highlighting the role of post-treatment methods in optimizing membrane performance.

Complementary to those presented, synergistic crosslinking with agents like glutaraldehyde and ammonium persulfate represents a new and innovative strategy for tailoring the performance of hydrophilic polymers in diverse applications. This method enhances the structural complexity of PVA membranes, significantly improving water resistance, thermal stability, and swelling control [80]. A green fabrication process, which is derived from renewable and biodegradable materials, offers a promising solution to environmental concerns associated with conventional membrane fabrication. For example, the use of citric acid and succinic acid as crosslinking agents in PVA membranes offers a more sustainable and environmentally safer alternative to conventional crosslinking agents (without introducing hazardous reagents), while improving membrane stability and durability [81].

These examples underscore the versatility of crosslinking techniques in tailoring PVA-based membranes for diverse applications, demonstrating their critical role in advancing membrane technology for environmental, biomedical, and industrial use.

### 3.2. Interfacial Polymerization

Interfacial polymerization (IP) is a widely used chemical technique for fabricating ultrathin, selective polymer films on porous supports. This method involves a reaction occurring at the interface between two immiscible phases: an aqueous phase containing a water-soluble monomer (e.g., amines such as m-phenylenediamine) and an organic phase with a water-immiscible reactive compound (e.g., oil-soluble monomers like trimesoyl chloride in a nonpolar solvent such as hexane) [82,83]. When these two solutions come into contact, a rapid polymerization reaction takes place at the interface, forming an ultrathin crosslinked polymer layer over the porous substrate. Typically ranging from 10 to 200 nm, this ensures high selectivity [84,85]. Membranes produced through interfacial polymerization have been extensively applied in high-rejection filtration processes, particularly in desalination, where they efficiently remove salts due to their exceptional selectivity and performance. Their properties, such as permeability, rejection rate, and fouling resistance, can be finely tuned by adjusting the monomer selection, reaction conditions, or incorporated additives. Moreover, IP membranes are highly scalable, making them suitable for large-scale production in water treatment and industrial separation [8,86].

Despite these advantages, interfacial polymerization has certain limitations. The use of immiscible solvents, often required for the process, poses environmental concerns, restricting solvent choices. Additionally, the reaction occurs extremely rapidly, making it challenging to precisely control the polymer layer’s thickness and uniformity. Some IP membranes are also prone to fouling, necessitating surface modifications to enhance their long-term stability and efficiency. A promising approach to overcoming these challenges is the formation of crosslinked PVA films on various supports, leveraging PVA’s intrinsic hydrophilicity to improve membrane performance. For instance, Miao et al. [87] developed poly(vinyl alcohol)/carboxymethyl cellulose sodium blend composite nanofiltration membranes via interfacial polymerization. Their study demonstrated that the incorporation of PVA enhanced membrane hydrophilicity and selectivity while improving antifouling properties. Such advancements highlight the significant impact of interfacial polymerization in tailoring membrane characteristics for diverse applications, particularly in water purification and separation technologies.

### 3.3. Grafting

**Figure 4:**
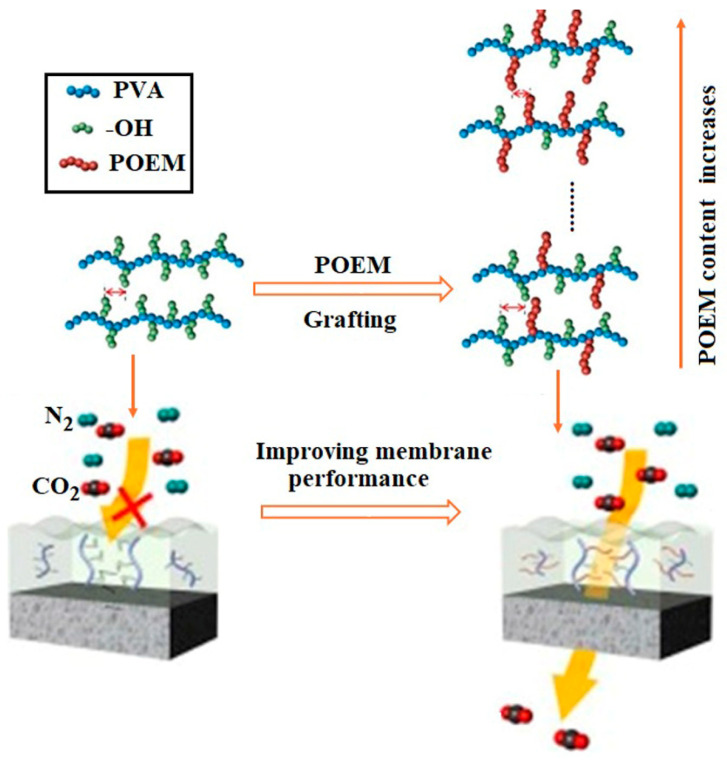
The method to obtain permeable PVA-g-POEM membranes with improved performance in gas-separation processes, using the grafting technique (graft copolymer consisting of PVA main chains and poly(oxyethylene methacrylate) (POEM) side chains). Adapted with permission from [91].

Grafting is a versatile chemical modification technique used to introduce functional groups onto a polymer backbone, significantly enhancing properties such as hydrophilicity, antifouling resistance, mechanical strength, and chemical stability. In the case of PVA-based membranes, grafting not only improves water resistance, selective permeability, and biocompatibility but also enhances chlorine resistance, antifouling properties, mechanical strength, thermal stability, hydrophilicity, electrical conductivity, flame retardance, and transparency [88,89,90,91]. In this context, Kim et al. [91] synthesized PVA-g-POEM graft copolymers for highly permeable thin-film composite membranes, demonstrating the effectiveness of grafting in enhancing membrane performance (Figure 4).

Grafting can occur either throughout the material (bulk grafting) or exclusively on the surface (surface grafting). Bulk grafting typically involves free radical polymerization, where initiators such as benzoyl peroxide (BPO), ammonium persulfate (APS), or azobisisobutyronitrile (AIBN) generate radicals on the PVA backbone. These radicals react with monomers such as acrylic acid or acrylamide, leading to the covalent attachment of functional groups. This approach enhances the overall structural integrity of PVA membranes while improving their functional properties. In contrast, surface grafting is a targeted technique that modifies only the outermost layer of a polymer without altering its bulk properties. This method is particularly valuable for applications where surface interactions are critical, such as filtration, biomedical implants, and antifouling coatings [92,93]. Various surface grafting strategies include photo-induced grafting, plasma grafting, radiation-induced grafting, thermally induced grafting, and ozone-induced grafting [94]. Additionally, enzymatic grafting, using enzymes like laccase or peroxidase, enables the attachment of bio-functional molecules onto PVA surfaces [95,96,97]. By enabling the covalent attachment of diverse vinyl monomers, surface grafting effectively enhances membrane hydrophilicity, biocompatibility, and antifouling resistance [98], while preserving the original mechanical structure. This approach has proven innovative and highly effective in improving the performance of PVA-based membranes, particularly for applications requiring optimized surface properties.

Advances in PVA membrane fabrication emphasize environmentally friendly approaches, such as bulk modification—introducing fullerenol and poly(allylamine hydrochloride)—combined with surface functionalization through polyelectrolyte multilayers. The method enhances permeability and selectivity by using water as the processing medium, while maintaining an environmentally friendly approach [99]. The growing body of research on grafting methods underscores their critical role in advancing membrane technology for filtration, biomedical, and industrial applications.

### 3.4. Blending with Other Polymers

Blending PVA with other polymers or compounds is a widely used approach to enhance membrane performance, offering improvements in mechanical strength, selectivity, thermal stability, permeability, and antifouling properties. This method allows for the development of membranes tailored for specific applications by incorporating hydrophilic or hydrophobic polymers, such as polyacrylic acid (PAA), chitosan, and polyethylene glycol (PEG), as well as nanoparticles like TiO_2_ and graphene oxide. For example, Cheng et al. [100] developed graphene oxide/PVA composite membranes, demonstrating enhanced pervaporation performance for ethanol dehydration due to improved structural integrity and selectivity.

The impact of polymer blending on membrane morphology and performance has been explored in several studies. This aspect was highlighted by Chuang et al. [101], who investigated asymmetric PVA membranes (Mw = 74,800 g/mol), incorporating dextran and poly(vinyl pyrrolidone) (PVP) into the casting solution. Using water as the solvent and Na_2_SO_4_/KOH/H_2_O as the coagulant medium, the researchers observed that PVP promoted the formation of compact structures, whereas dextran facilitated pore formation on the membrane’s top layer. The modified membranes were evaluated for their ultrafiltration capabilities, specifically regarding the rejection of dextran and PVP, highlighting the role of polymer additives in tuning membrane porosity and separation efficiency.

Thus, by blending PVA with different polymers and nanomaterials, researchers have successfully enhanced, on the one hand, the membrane’s performance in various applications, from pervaporation and ultrafiltration to biomedical and industrial uses. The ability to fine-tune properties such as porosity, hydrophilicity, and mechanical strength through blending underscores the significance of this technique in advancing PVA-based membrane technologies. On the other hand, the incorporation of bio-based additives or fillers is expected not only to enhance membrane properties, but also to provide new insights into the development of high-performance, eco-friendly PVA-based products with practical applications [102].

### 3.5. Phase Inversion Method

Phase inversion is a fundamental method for producing polymer membranes, recognized for its effectiveness in creating structures with well-defined porosity. This physical process is particularly important in developing PVA-based membranes, as it enables precise control over their morphology and performance [103]. It involves inducing phase separation in a homogeneous polymer solution to create a solid membrane with controlled porosity. The polymer-rich phase forms the membrane’s solid structure, while the polymer-lean phase contributes to pore formation [104]. The selection of solvent and coagulation conditions plays a crucial role in determining membrane morphology and performance.

Phase inversion can occur through various mechanisms, including the following:

1. *Immersion Precipitation (Non-Solvent Induced Phase Separation—NIPS):* NIPS is the most widely employed technique, pioneered by Loeb and Sourirajan, initially for seawater desalination [105]. Originally developed for seawater desalination, it enabled the fabrication of the first cellulose acetate membrane and remains one of the most established and extensively used approaches for polymeric membrane synthesis. This method involves immersing a polymer solution in a non-solvent bath, leading to rapid phase separation and the formation of asymmetric membranes with distinct pore structures.

A comprehensive thermodynamic study prior to membrane fabrication is essential for determining the optimal processing parameters, thereby minimizing experimental uncertainties and enhancing membrane performance [106]. Mathematical modeling plays a crucial role in this regard, by providing a deeper understanding of the membrane formation process mechanisms. Thus, to construct a rigorous phase inversion model, two fundamental sets of equations must be considered: thermodynamic equations—which describe the equilibrium conditions at the interface of the two phases, establishing criteria for different thermodynamic regions with high accuracy—and kinetic equations which encompass conservation laws governing mass transport of components until the thermodynamic boundaries are reached [107].

**Figure 5:**
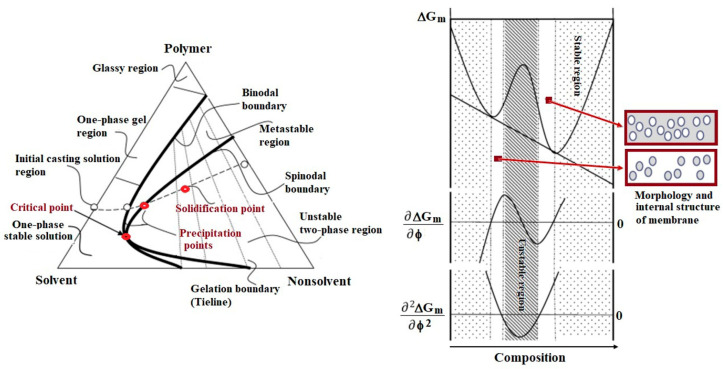
Thermodynamic regions illustrated in phase diagram for a ternary system and the dynamic evolution of compositions, using the Gibbs free energy equation, which finally results in different morphologies and internal structure of membranes. Adapted with permission from [106].

A representative example of this approach is presented in the work of Keshavarz et al. [106]. The authors conducted an extensive thermodynamic study to formulate and validate thermodynamic equations for constructing ternary phase diagrams based on Gibbs free energy. Their work provides a more precise and predictive framework for understanding membrane formation, offering deeper insights into phase inversion mechanisms and optimizing membrane properties. In their study, Keshavarz et al. have defined key phase boundaries, including binodal and spinodal limits, which dictate phase-separation pathways. Beyond these boundaries, they incorporated appropriate kinetic formulations to account for the instantaneous thermodynamic state of the solution, as determined by the thermodynamic model. This process is commonly visualized using ternary phase diagrams, as illustrated in Figure 5, which represent the equilibrium and phase-separation behavior of polymeric solutions.

By utilizing eco-friendly materials, the toxicity footprint of membrane fabrication techniques can be considerably reduced. For example, the transformation of the conventional non-solvent induced phase-separation method into an environmentally sustainable process is possible by integrating renewable and biodegradable materials. The innovative approach involves utilizing polylactic acid (PLA) as a biodegradable polymeric base, dimethyl sulfoxide (DMSO) as a green solvent, and deep eutectic solvents (DES) as eco-friendly additives [108]. These substitutions significantly reduce the toxicity footprint associated with membrane fabrication while addressing environmental concerns. Through this exemplification, we want to emphasize that, from an environmental perspective, green membrane manufacturing can simultaneously improve performance and sustainability, paving the way for eco-friendly industrial applications while reducing hazardous waste and dependence on toxic chemicals.

2. *Evaporation-Induced Phase Separation (EIPS):* EIPs is commonly used for PVA-based membranes, where solvent evaporation drives phase separation. Solvent selection, temperature, humidity, and volatility influence the membrane’s morphology. Typically, water or water–alcohol mixtures are employed to produce dense, defect-free membranes, suitable for applications like pervaporation and gas separation. However, due to PVA’s water solubility, chemical crosslinking (e.g., glutaraldehyde or boric acid) or thermal treatment is required for stability [109].
3. *Vapor-Induced Phase Separation (VIPS):* First introduced by Zsigmondy and Bachmann [110] and later advanced by Elford [111], this technique involves exposing the polymer solution to a vapor environment, leading to solvent diffusion into the vapor phase while non-solvent diffuses into the polymer. Plasticizers or crosslinkers (e.g., boric acid) enhance membrane flexibility and stability, making this method suitable for water filtration, biosensors, and drug delivery [21,112,113,114].
4. *Thermally Induced Phase Separation (TIPS):* TIPS involves cooling a polymer solution, causing the polymer to precipitate as its solubility decreases. PVA membranes produced by this method exhibit porous structures with controlled pore sizes, essential for selective filtration and high permeability applications. Common solvents include water and dimethyl sulfoxide (DMSO), with added non-solvents to regulate phase separation.

**Figure 6:**
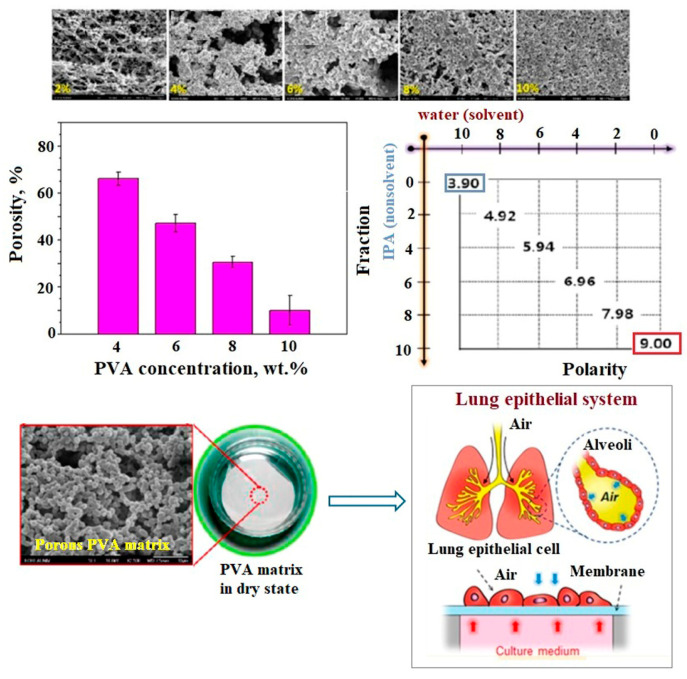
Schematic diagram illustrating the fabrication of a porous poly(vinyl alcohol) matrix as a functional model of the lung epithelial system: SEM images and porosity of the matrix as a function of the PVA solution concentration; theoretical polarity of the cosolvent mixtures. Adapted with permission from [115].

Over time, researchers have focused on demonstrating the effectiveness of phase inversion techniques in the fabrication of high-performance PVA membranes. To this end, numerous studies have explored various fabrication approaches. For example, Chae et al. [115] developed a porous PVA membrane using water as the solvent and isopropyl alcohol as the non-solvent, demonstrating its potential as an extracellular matrix. The controlled crystallization process, driven by solute precipitation and dissolution in the ternary solvent system, led to the formation of packed microspheres with tunable porosity ranging from 67.6% to 14.9%, depending on polymer concentration. In low-polarity solvents, self-aggregation of PVA chains occurred through dipole–dipole interactions, significantly influencing the membrane’s structural properties. The resulting thin and porous PVA matrix effectively mimicked the pulmonary epithelial environment, serving as a functional extracellular matrix (Figure 6).

Following the same idea, Kim and Lee [116] fabricated integrally skinned asymmetric PVA membranes (99+% hydrolyzed, Mw = 31,000–51,000 g/mol) using a combination of N-methyl-2-pyrrolidone (NMP) and water as cosolvents, with 2-propanol as the non-solvent. The PVA solution (10 wt.%) was prepared at 80 °C for 12 h, cast at 25 °C, and immersed in isopropanol for 20 min. The membrane structure transitioned from dense to asymmetric depending on the NMP/water ratio. Instead, Ahmad et al. [117] developed asymmetric PVA membranes (88% hydrolyzed, Mw = 88,000 g/mol) using deionized water as the solvent and a coagulant mixture of sodium hydroxide and sodium sulfate (1:2). Post-fabrication, the membranes were crosslinked with glutaraldehyde, sodium sulfate, and sulfuric acid for different durations (0.5–2 h). SEM analysis showed that increasing crosslinking time resulted in a narrower pore size distribution, affecting hydrophilicity and water flux.

According to the mentioned studies, it can be stated that the phase inversion method is a versatile and effective technique for producing polymeric membranes with tailored structures and functionalities. By carefully selecting solvents, non-solvents, and processing conditions, researchers can optimize membrane properties for diverse applications. PVA-based membranes, in particular, benefit from different phase inversion approaches, allowing for fine control over porosity, mechanical stability, and chemical resistance.

### 3.6. Dip-Coating

Dip-coating is a physical deposition technique employed for fabricating PVA-based membranes, offering a straightforward and efficient approach to achieving thin, uniform coatings. This method involves immersing a support layer, such as a porous membrane or nonwoven fabric, into a dilute polymer solution bath (e.g., PVA), followed by crosslinking and drying through controlled heat treatment [118]. During crosslinking, chemical agents enhance the adhesion between the deposited PVA layer and the underlying support, improving the mechanical stability and water resistance of the final membrane [119]. Once the coated membrane is removed from the polymer bath, a thin, uniform film forms on the substrate. The solvent is subsequently eliminated through evaporation, ensuring proper film formation and adhesion [120].

The dip-coating method plays a crucial role in the fabrication of PVA-based membranes, particularly in achieving controlled thickness, uniformity, and functional surface properties. Studies have demonstrated that dip-coated membranes exhibit improved performance characteristics, making this technique highly valuable across multiple applications. For example, Tang and Yan [118] explored dip-coating for fibrous materials, emphasizing its mechanism, various deposition methods, and applications in membrane fabrication. Their study highlights how dip-coating enables tailored coatings with precise surface modifications, making it an essential method for developing functionalized membranes. Additionally, Himma et al. [121] investigated the influence of non-solvents on the surface properties, in terms of the morphology and hydrophobicity of dip-coated polypropylene membranes. Their findings demonstrate how solvent composition directly affects membrane structure, enabling the fine-tuning of surface wettability and porosity to meet specific application needs.

Therefore, dip-coating is a scalable, cost-effective method for fabricating thin-film composite membranes, biomedical coatings, and protective layers. In particular, PVA-based membranes produced via dip-coating are used in water filtration, pervaporation, and biomedical applications due to their high hydrophilicity and selective permeability. This technique ensures uniform, defect-free coatings on porous supports while allowing precise control over the membrane thickness and functional properties, making it a versatile approach for various industrial applications.

### 3.7. Electrospinning

Electrospinning is an advanced technique for fabricating nanofibrous membranes with a high surface area and porosity, making it particularly suitable for applications in filtration, desalination, and biomedical fields [122,123]. Originally introduced by Formhals as a method for processing textile yarns [124], electrospinning has since evolved into a key manufacturing approach for a wide range of applications, including medical materials, protective textiles, and filtration membranes [125,126,127]. In this process, a high voltage is applied between a polymer solution droplet and a grounded collector, inducing electrostatic forces that overcome surface tension, resulting in the formation of ultrafine fibers [122]. The fiber morphology, including diameter and arrangement, significantly influences the porosity, pore size distribution, and hydrophilicity of electrospun membranes, allowing for the precise tailoring of properties such as air permeability.

PVA is commonly used in electrospinning; while PVA with a low degree of hydrolysis (DH), such as 88%, is frequently employed, electrospinning highly hydrolyzed PVA (>98% DH) presents challenges due to variations in molecular weight and solvent properties. Optimizing the polymer solution composition is crucial to achieving stable electrospun structures [128,129,130]. Additionally, due to PVA’s hydrophilic nature and tendency to form hydrogels, further stabilization techniques are required to maintain fiber integrity in aqueous environments. Various strategies, including physical and chemical crosslinking, have been explored [131,132,133]. An example in this sense is given by the studies conducted by Wang et al. [134] who investigated highly porous electrospun PVA membranes (using PVA with different degrees of hydrolysis) crosslinked with glutaraldehyde (GA) in acetone for oil/water separation. Their ultrafiltration tests on PVA-coated scaffolds with a hydrogel layer (~1.8 µm) demonstrated a significantly higher water flux (130 LMH) compared to Pebax 1074-coated membranes (57 LMH), underscoring the advantages of electrospun PVA membranes in separation processes.

To improve stability without toxic crosslinkers, citric acid has been proposed as an alternative. Truong et al. [135] conducted a comparative study on the crosslinking stability of PVA using citric acid, maleic acid, and poly(acrylic acid) (PAA) for applications, such as metal ion uptake and ammonia adsorption, demonstrating the potential of citric acid as a viable crosslinking agent [136].

On the other hand, in biomedical applications, minimizing toxic additives, such as chemical crosslinking agents, is essential for ensuring biocompatibility and safety for human use. Thermal treatment is a promising alternative to chemical crosslinking, enabling polymer stabilization without introducing potentially harmful agents [132,133]. This method not only helps maintain the morphology of electrospun mats but also preserves the free hydroxyl groups on the material’s surface, which are crucial for the attachment of immunoaffinity ligands [137].

**Figure 7:**
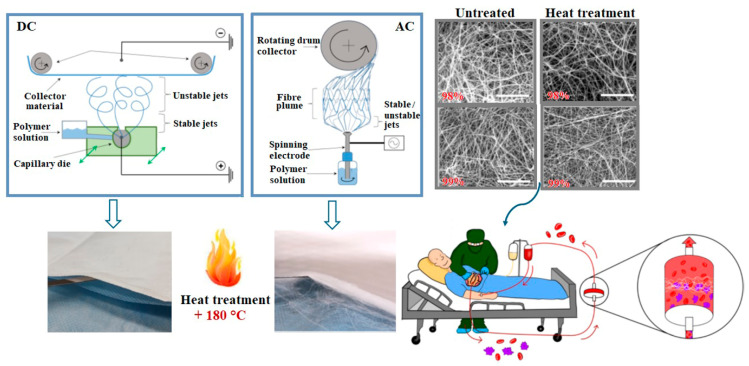
Schematic representation of steps taken towards creating an alternative platform for removing tumor cells before they are returned to the patient: obtaining nonwoven membranes by electrospinning (wire-based needleless DC electrospinning and AC electrospinning with a rod-like electrode and rotating drum collector) and heat treatment at 180 °C, using PVA 98% and 99% hydrolyzed. SEM images are shown of electrospun PVA membranes with 98% and 99% DH, obtained by DC electrospinning, designed for use in the cell salvage process. Adapted with permission from [138].

Based on this hypothesis, Homer et al. [138] successfully fabricated non-woven PVA membranes via electrospinning, followed by heat treatment at 180 °C to enhance water resistance and preserve fibrous morphology. Their study demonstrated that 99% hydrolyzed PVA mats processed using DC electrospinning exhibited superior mechanical strength and water resistance compared to 98% DH counterparts, while maintaining cytocompatibility (Figure 7).

**Figure 8:**
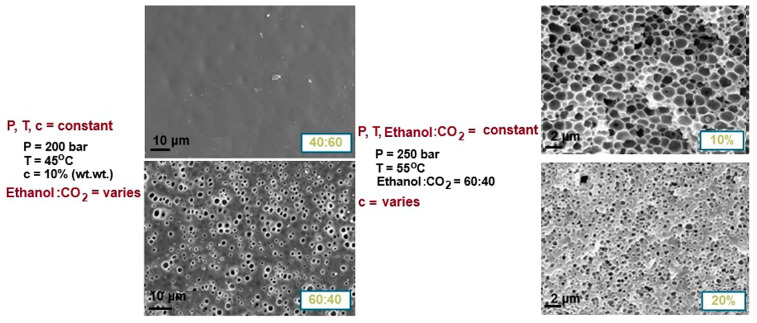
Effect of ethanol/CO_2_ ratio and polymer solution concentration on the morphology of PVA membranes. Adapted with permission from [141].

In the attempt to create porous structures, particularly PVA-based membranes, various innovative approaches have been explored, leading to significant discoveries that demonstrate their impact on membrane structure and properties. One interesting technique involves the use of microfluidics, which has shown promise in creating precise and controlled porous structures in polymer membranes [139]. Another innovative approach utilizes supercritical CO_2_ as a component in polymeric solutions [140]. In their studies, Reverchon et al. [141] explored the preparation of PVA membranes through supercritical CO_2_-assisted phase inversion. Their research focused on key preparation parameters, including the polymer concentration, ethanol/CO_2_ ratio, temperature, and pressure. They were able to achieve various morphologies, with macropores ranging from 0.5 to 4 μm and a top layer that could either be dense or porous, as illustrated in Figure 8.

Another promising method for fabricating PVA membranes is gas foaming, as demonstrated by Narkkun et al. [142]. They utilized CO_2_ generated from the thermal decomposition of sodium bicarbonate (NaHCO_3_) at 130 °C to produce L-arginine functionalized PVA membranes with an average molecular weight of 130,000 g/mol and 99% hydrolysis. This method resulted in membranes with an average pore size ranging from 32 to 56 μm, depending on the amount of grafted L-arginine.

In conclusion, PVA-based membranes can be fabricated using diverse chemical and physical methods, each offering distinct advantages depending on the intended application. The various approaches discussed demonstrate how different fabrication techniques influence membrane properties, such as pore size and surface morphology, ultimately determining their suitability for specific uses. The continuous evolution of these methods plays a crucial role in advancing high-performance membranes for applications in water purification, biomedical engineering, and industry.

## 4. Environmental Applications of PVA-Based Membranes

Population growth and anthropogenic activities lead to an increase in the consumption of energy and pure water. Membrane-based processes represent a sustainable solution considering the growing need for energy and water. Membranes play an essential role in fuel cells, for energy generation, but also in membrane processes for water filtration and purification, acting as a semi-permeable barrier that allows the passage of certain species and restricts others. When designing a membrane, it is always necessary to take into account the applicability domain, target species, operating principle, and feed stream and its composition, as well as expected results. Thus, depending on these factors, the structure, morphology, and chemical functionality of the membrane will be predetermined. The structural properties will be those that reflect the chemical, mechanical, and dimensional stability, while the functional properties will give the selectivity and permeability.

### 4.1. PVA-Based Membranes for Fuel Cell

Generally, the membranes used in fuel cells are obtained from an electrolyte polymer, with high conductivity, optimal water sorption capacity, gas permeability, and high mechanical resistance [19,20,143]. In addition to the polymers recommended in processing membranes used in this sense (e.g., cationic or anionic functionalized polymers, sulfonated aromatic polymers or quaternized ones), researchers have focused on finding new directions for obtaining advanced materials that present higher efficiency in energy generation and a relatively low cost. In this regard, they have paid more attention to PVA mainly due to its film-forming property and relatively low cost. The hydroxyl groups will generate strong hydrogen bonds that will give it strong methanol resistance, with PVA being recommended for obtaining membranes used in direct methanol applications and alkaline fuel cell applications [77]. Molla and collaborators [144] have obtained novel Nafion/PVA ultrathin composite proton exchange membranes (PEMs) with a thickness varying between 19 and 60 μm through electrospinning. They tested them in terms of mechanical strength, proton conductivity, and efficiency in fuel cells. The results showed that the tensile strength and Young’s modulus are higher than for Nafion membranes, while the proton conductivity varies nonuniformly. Low conductivity was explained by non-ionic conductivity features induced by the PVA phase. However, the membranes with a thickness of less than 20 μm presented high efficiency in fuel cell operation.

In the same context, Mohammed et al. [145] have achieved new proton exchange membranes (PEMs) based on polyvinyl alcohol (PVA) and sulfophthalic acid (sPTA) through a casting and crosslinking method, varying the sPTA content in systems from 10 to 30 wt.%. The results showed that increasing the sPTA content led to an increase in thermal stability, an important characteristic for fuel cells. The membranes used for this purpose should have a high thermal stability; otherwise, hot spots may appear, leading to membrane aridity and cracking. Moreover, the dielectric constant and ionic conductivity increased with increasing of sPTA content, highlighting its role in improving the membranes properties for use in the intended application.

**Figure 9:**
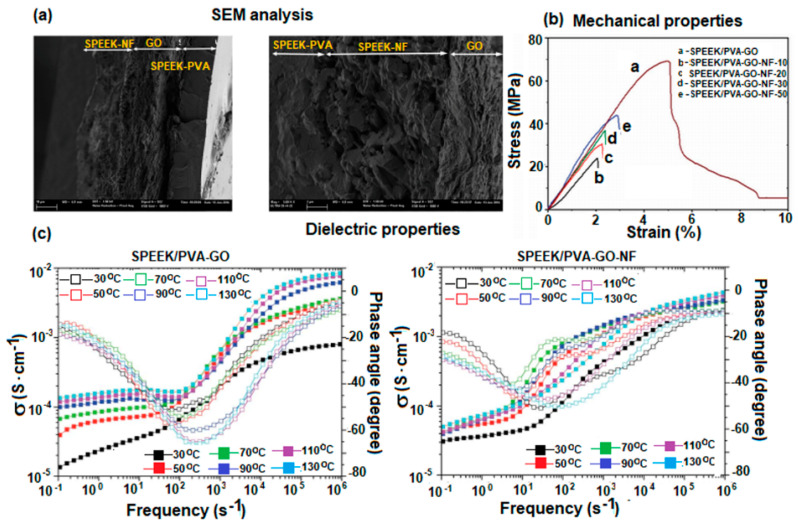
SEM images of SPEEK/PVA/GO-NF membranes (**a**) mechanical properties (**b**) proton conductivity and phase angle on frequency (**c**) for SPEEK/PVA-GO and SPEEK/PVA-GO-NF composite membranes at different temperatures. Adapted with permission from [146].

Other studies on PVA-based composite materials for obtaining PEMs have also been reported in the literature. For instance, two series of composite membranes based on sulfonated poly(ether ketone) (SPEEK)/polyvinyl alcohol (PVA)/graphene oxide (GO) were made [146]. A series (SPEEK/PVA/GO) containing a GO layer sandwiched between a SPEEK/PVA matrix, and another (SPEEK/PVA/GO-NF) consisting of SPEEK/polyvinyl butyral (PVB) nanofibers sandwiched between two layers of GO were produced. The three layers were sandwiched, in turn, in a SPEEK/PVA phase matrix. The results of the performed tests showed that the proton conductivities of the SPEEK/PVA/GO membranes increased with temperature, from ${1\times 10}^{-3}\;S\cdot {cm}^{-1}\;$for 30 °C to ${8.3\times 10}^{-3}\;S\cdot {cm}^{-1}$ for 130 °C, being higher than those for SPEEK/PVA/GO-NF (Figure 9).

Analyzing the data as a function of fiber thickness, the researchers concluded that the conductivity values increase with nanofiber thickness for all temperatures. Also, the findings highlight the importance of the GO layer on the membrane performance for high temperatures.

Another study realized by Sahin [147] consisted of obtaining of new type of membrane based on sulfonated poly ether ether ketone (SPEEK)/PVA and tetraethyl orthosilicate (TEOS), by a blending and casting method. Studies have shown that, in addition to the fact that PVA facilitates the incorporation of TEOS into the system, it also increases the membrane’s mechanical strength. The presence of TEOS leads to a reduction in the membrane water loss at high temperatures, an increase in proton conductivity, and implicitly, an improvement in fuel cell performance. Maarouf et al. [148] have achieved novel ionic polymer membranes based on polyvinylpyrrolidone (PVP), PVA, sulfosuccinic acid, and silicotungstic acid (SiWA), with and without silica. The results of the studies showed that the obtained composite membranes are stable at temperatures exceeding 180 °C and present higher values of the ion proton conductivities compared with the Nafion112 commercial membrane and, therefore, they can be successfully used as PEMFCs.

**Figure 10:**
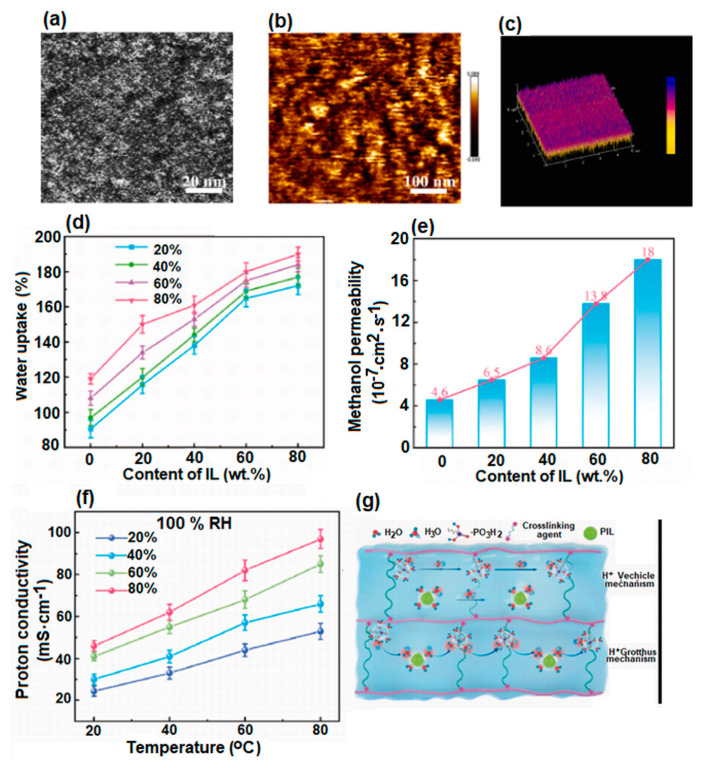
(**a**) TEM images, (**b**,**c**) 2D and 3D AFM phase diagram of CPVA/PIL-20 membrane, (**d**) water uptake, (**e**) methanol permeability, (**f**) proton conductivity, and (**g**) schematic diagram of the proton conduction mechanism of CPVA/PILs membranes. Adapted with permission from [149].

Also, taking into account the relatively high costs of commonly used membranes (Nafion membranes) for direct methanol fuel cells, as well as their high methanol permeability, the investigations continued with the aim to design materials to be as cheap as possible, with improved mechanical and thermal resistance, optimal proton conductivity, and minimal methanol permeability. PVA is recommended for obtaining membranes due to the above-mentioned properties (optimal flexibility and film-forming capacity), but it has low conductivity. This inconvenience was overcome by Pan et al. [149] via processing composite membranes crosslinked with PVA (CPVA) and doped with a highly conductive proton-ionic liquid (PIL) (Figure 10).

The latter brings a surplus of protons to the composite membrane, directly participating in the proton transport within the membrane. The researchers have used three types of ionic liquids ([Dema][TfO], [EIm][TfO], and [2-Sema][TfO]) and selected the one with the best performance ([Dema][TfO]). This was doped into the crosslinked system with PVA, leading to a surplus of conductive groups with an impact on membrane conductivity. The data obtained have shown that the composite membranes doped with ionic liquids exhibit improved mechanical strength, thermal stability around 200 °C, and enhanced ion exchange capacity and proton conductivity. Furthermore, the CPVA-80% PIL membrane permeability for methanol is four times higher than that of the control CPVA membrane without PIL. The studies carried out have led to the conclusion that membranes with a content of 20% PIL exhibit the optimal properties to be successfully used in the field of direct methanol fuel cells. Elerian et al. have obtained membranes based on a crosslinked poly(vinyl alcohol)/5-sulfosalicylic acid dehydrate (PVA/SSCA) composite [150]. Different types of crosslinking agents (glutaraldehyde (GA), fumaric acid (FA), and malic acid (MA)) were introduced into the PVA/SSCA mixture in order to chemically restrict the formed network. The results of the studies demonstrated what was expected: the presence of SSCA in the system led to an improvement in proton conductivity, while crosslinking agents led to an increase in chemical stability and mechanical and water resistance. Increasing the SSCA content leads to an increase in the ion exchange capacity (IEC), with the proton conductivity being comparable to that reported in the literature for other PVA composite membranes. In addition, the methanol permeability was determined to be lower than for the Nafion117 membrane, making it a good candidate for application as a membrane for direct methanol fuel cells.

For the same purpose, composite membranes based on PVA, titanium dioxide (TiO_2_), and carbon nanoparticles (NPs) have also been synthesized by Kalid and co-workers [151]. TiO_2_ and carbon NPs were introduced into the matrix with the aim of improving the mechanical and thermal properties. Subsequent treatment of the membranes with maleic acid (crosslinking agent) leads to an improvement in the electrochemical properties. The obtained results showed that both thermal stability and mechanical strength were improved. Moreover, the composite membranes presented optimal conductivity and the lowest methanol permeability generated by the intercalation of TiO_2_ NPs, making them good candidates for the intended application.

### 4.2. PVA-Based Membranes for Water Treatment

Biological pollution and low groundwater levels are two of the global problems posing a threat to both humans and nature. It is expected that due to population growth, in 2025, half of the world’s population will live in areas affected by water shortages. For this reason, researchers have focused on membrane technology development, because it plays an essential role in the treatment and production of drinking water [152,153,154]. Water treatment processes involve different types of membranes, with different properties, used to remove various contaminants and produce clean water. There are four types of membranes used in this process: microfiltration, ultrafiltration, nanofiltration, and reverse osmosis. PVA-based membranes are most often used in this regard because their hydrophilic character leads to an increase in membrane permeability. However, the weak mechanical properties, low water resistance, and fouling tendency of PVA membranes remain real challenges for researchers in terms of their use for water treatment and purification [155,156]. For this reason, recently, research studies were conducted for the improvement of these properties through methods such as chemical crosslinking, mixing, creation of nanocomposites, or thermal treatment. Below, some of the most current studies conducted on PVA membranes in order to improve their performance in water treatment and purification applications are presented.

#### 4.2.1. PVA-Based Microfiltration Membranes (MF)

**Figure 11:**
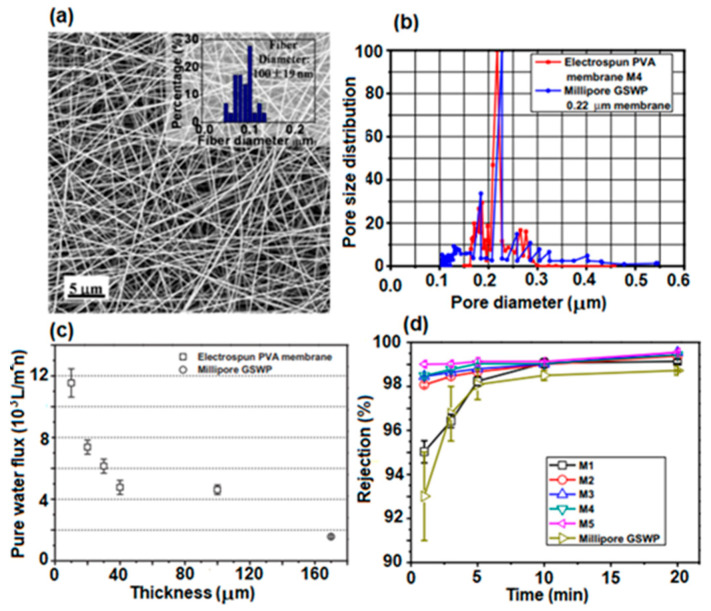
SEM images of membranes obtained by electrospinning of PVA solution of 10 wt.% (**a**), pore size distribution (**b**), water flux through the membrane (**c**), and rejection of polycarboxylate microspheres (**d**) for the electrospun PVA membranes of different thicknesses and Millipore GSWP 0.22 μm. Adapted with permission from [158].

Microfiltration membranes are porous membranes with pore sizes ranging between 0.1 and 5.0 μm, capable of separating large suspended solids (colloids, particulates), fat, bacteria, algae, or protozoans from contaminated water with an efficiency of 99.9% [157]. For complete water treatment, they are usually used in combination with ultrafiltration membranes. Microfiltration membranes with the possibility of use in this direction were obtained by Liu et al. [158] (Figure 11). The MF membranes consisted of an electrospun PVA membrane that was deposited on a non-woven composite substrate of polyethylene terephthalate (PET), followed by chemical crosslinking with glutaraldehyde (GA), in acetone.

Structural characterizations of the obtained membranes showed that those with a thickness of 20 μm have an average pore size of 0.24 μm, comparable with that of the Millipore GSWP 0.22 μm membrane. However, due to the high porosity, the newly synthesized ones have a water flux of 3 to 7 times higher than that of the reference membrane. In addition, the results of the tests achieved on a suspension of polycarboxylate microsphere particles of 0.20 μm in diameter showed a removal rate of 98%. The processed membranes have a high efficiency considering the “cake structure” formed during the removal tests and the fact that they maintain a permeation flux of approximately 1.5–6 times higher than that of the standard, so that they can be recommended for the water treatment and purification applications. Recently, inorganic nanoparticles have started to be used on a large scale in the development of high-performance membranes for MF, UF, RO, pervaporation, and gas-separation processes. Metal oxides as zinc oxide (ZnO) and titanium dioxide (TiO_2_) are widely utilized as inorganic fillers in separation membranes [159,160]. The mechanical properties, water resistance, and antifouling performance of PVA membranes were improved by addition into a polymeric matrix of TiO_2_ nanoparticles. Karimi and collaborators have obtained photo-crosslinked PVA fibrous membranes, using citric acid as a crosslinking agent and TiO_2_ as a photocatalyst [161]. The crosslinking reaction took place in the presence of TiO_2_ under UV irradiation; an increase in photocatalyst content leads to the formation of well-weaved networks of thin fibers with high hydrophilicity. Microfiltration tests (consisting of separation of oil from water emulsions) have shown that the PVA/TiO_2_ nanofibers present a higher permeation flux and oil rejection, but a decrease in fouling tendency compared to the PVA membrane. The best separation performance was obtained for membranes containing 1 wt.% TiO_2_ nanoparticles. The results lead to the conclusion that the incorporation of TiO_2_ nanoparticles and UV irradiation improve the separation process and antifouling properties, with the method approached by the researchers being a more economical way than the common reticulation methods. Similar studies were carried out by Sakarkar et al. and Yeo et al. [162,163], who obtained membrane materials with enhanced hydrophilicity and improved dye rejection during filtration processes and the possibility of being reusable.

#### 4.2.2. PVA-Based Membranes for Nanofiltration Process (NF)

**Figure 12:**
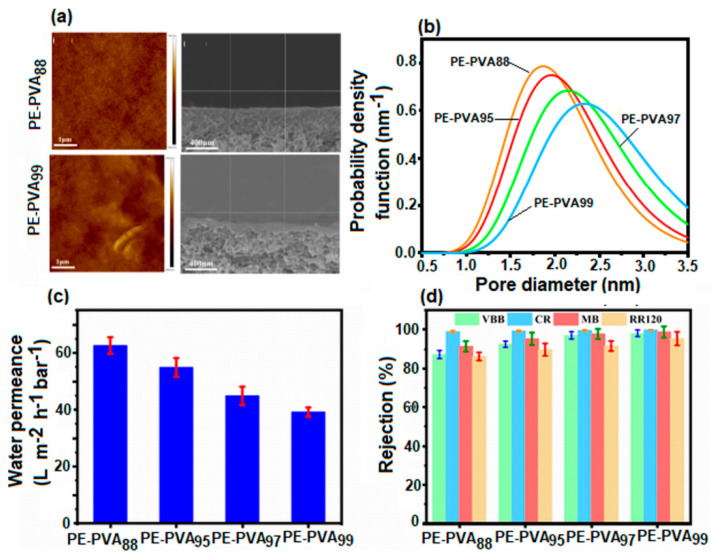
Two-dimensional AFM and SEM cross-section of PE-PVA88 and PE-PVA99 membranes (**a**), pore size distribution (**b**), water permeability (**c**), and dye rejection capacity (**d**) of PE-PVA membranes. Adapted with permission from [167].

The nanofiltration process represents a versatile process that takes place under pressure and allows the removal of dyes, organic molecules, and salt ions with a molecular weight between 200 and 1000 Da from wastewater. For efficiency, the membranes used in such processes should have a positively and negatively charged surface and the pore size should fall within the 1.0–10 nm range [164,165]. Polymers recommended for NF membranes are cellulose derivatives and biopolymers whose special characteristics are high water permeability, solute rejection, selectivity, water recovery, and flux. However, researchers have extended their studies with the aim of reducing the cost and improving membrane efficiency by increasing hydrophilicity, biofouling resistance, and selectivity. As mentioned, PVA has attracted attention due to its potential for membrane modification through grafting, crosslinking, and the inclusion of materials at the nanoscale. In this context, a new technique for obtaining nanofiltration membranes was approached by Lin et al. [166]. They obtained a 1 mL stock solution of graphene oxide (GO) that was introduced into a Sterlitech HP 4750 dead-end filtration cell, later adding over this 30 mL of PVA solution and 50 mL of water. The membrane was achieved via pressurized dead-end filtration. The results of practical tests realized using methylene blue (MB) and alizarin red S (ARS) showed that PVA-coated GO membranes have a dye removal capacity of >90%. In addition, after 144 h of filtration, it maintains its removal efficiency at greater than 80% for both small molecular compounds, which demonstrates a promising potential to be applied at the industrial level. Another way to obtain high-performance ultrafiltration membranes was addressed by Zhu and collaborators, based on the intrinsic characteristics of PVA with different degrees of alcoholysis (88, 95, 97, and 99% hydrolyzed) [167] (Figure 12).

For low degrees of alcoholysis, the rate of interfacial polymerization (IP) reactions is low and the resulting membranes have a thin active layer at the surface and high water permeation but a satisfactory degree of selectivity. In contrast, those obtained from PVA with high degrees of alcoholysis present thicker active layers with an increased rejection rate, low water permeation, and high selectivity. Among all the analyzed membranes, the best performance was determined for 97% hydrolyzed PVA. The studies represent a new way through which nanofiltration performance can be improved, namely by adjusting the PVA alcoholysis degree.

The limited resistance to pollutants and residual chlorine led Cai et al. to develop new types of polyvinyl alcohol (PVA)/polyamide (PA)/polyvinyl alcohol (PVA) NF membranes of the sandwich type [168]. These consisted of a PA layer sandwiched between two layers of PVA. The membranes were tested in terms of structure, morphology, efficiency in the separation process, and the removal of salt and dyes, but also resistance to the action of pollutants and residual chlorine. The experimental results showed that as the PVA concentration in the casting solution increases from 0.15% to 0.25%, the permeation flux decreases and the salt (Na_2_SO_4_) rejection rate increases, reaching a maximum of 96.62%. Furthermore, the PVA-0.20/PA/PVA-0.20 tri-layer membrane shows an improvement in water flux, approximately three-times greater than the control sample (obtained through interfacial polymerization using a polysulfone (PS) substrate and piperazine (PIP) solution). Also, the salt removal rate exceeds 96%, while for Congo Red (CR) and Victoria Blue B (VB) it is 99%. Regarding their resistance at the pollutants’ actions, it was established that after immersion in 1000 ppm sodium hypochlorite (NaClO) solution, for 4 h, the PVA/PA/PVA composite NF membrane presents a rate of salt and dye rejection that is slightly lower, while that of the standard membrane decreases drastically. Considering the obtained results, the researchers support the industrial-scale design of these types of membranes and their use for water softening and dyed wastewater treatment. Other studies on the synthesis of PVC/PVA membranes in order to enhance NF performance were conducted by Hermani et al. [169]. They opted for the introduction of amine-functionalized ordered mesoporous silica (APTES-SBA-15) nanoparticles in the polyvinyl alcohol (PVA) top layer of a polyvinylchloride (PVC)-based membrane. The obtained results showed that the presence in the membrane of APTES-SBA 15 nanoparticles (in a percentage of 0.50%) increases the hydrophilicity, improves the water flux, and determines a rejection of the organic carbon-based pollutants at a percentage of 91.41%.

#### 4.2.3. PVA-Based Membranes for Ultrafiltration Process (UF)

**Figure 13:**
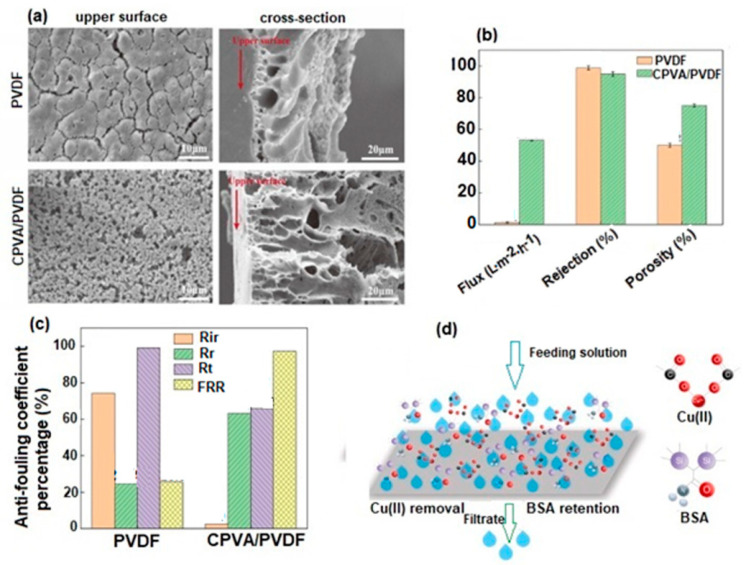
SEM images of the surface and cross-section of PVDF and CPVA/PVDF membranes (**a**), the permeate flux, rejection efficiency, and porosity of PVDF and CPVA/PVDF membranes (**b**), change in irreversible fouling (Rir), reversible fouling (Rr), total fouling (Rt), and fouling recovery rate (FRR) (**c**), and schematic representation of the efficiency of Cu(II) and bovine serum albumin (BSA) removal rate of PVDF and CPVA/PVDF membranes (**d**). Adapted with permission from [170].

The ultrafiltration processes involve the use of porous membranes, with an average pore size ranging between 0.01 and 0.1 μm, which allows the removal of most organic molecules, viruses, and salts. Chen and collaborators synthesized membranes based on carboxymethyl polyvinyl alcohol (CPVA) and PVDF for Cu (II) removal from water [170] (Figure 13). To increase the membrane performance (antifouling properties and capacity to remove both the organic macromolecules and ions from water), the group of researchers initially resorted to the chemical grafting of PVA and obtainment of carboxymethyl polyvinyl alcohol (CPVA). Subsequently, CPVA/PVDF ultrafiltration membranes were designed by the non-solvent co-induced phase-separation (NIPS) method.

Studies have shown that the composite membranes exhibit improved permeation flux compared to PVDF membranes and a removal capacity of bovine serum albumin (BSA) and Cu(II) of 92.00% and 90.89%, respectively, maintaining a high removal rate even after 10 cycles of sieving and adsorption. In addition, the data show a fouling recovery rate of 97.4%, which proves a good antifouling property. The obtained data provide a theoretical foundation for the application of CPVA/PVDF ultrafiltration membranes in wastewater treatment.

Also, membrane-based polyether sulfone (PES)/polyethylene glycol (PEG)/polyvinyl alcohol (PVA)/silicon dioxide (SiO_2_) nanoparticles with potential applications in water treatment and purification were synthesized by Widiyanti et al. [171]. PES/PEG/PVA/SiO_2_ composite membranes with the constituent components in different mixing ratios were obtained by the non-solvent induced phase inversion method. Variation in the system components’ composition leads to morphological and structural changes with an impact on the membrane permeability and selectivity in ultrafiltration processes. The study’s results showed that 17.25% PES, 3.72% PEG, 0.85% PVA, and 0.35% SiO_2_ represents an optimal composition that improves the membrane hydrophilicity, reducing fouling and enhancing water permeability. Consequently, the presence of SiO_2_ contributes to an increase in mechanical resistance and durability of the synthesized membranes, making them good candidates for wastewater treatment processes.

#### 4.2.4. PVA-Based Membranes for Reverse Osmosis (RO) Process

**Figure 14:**
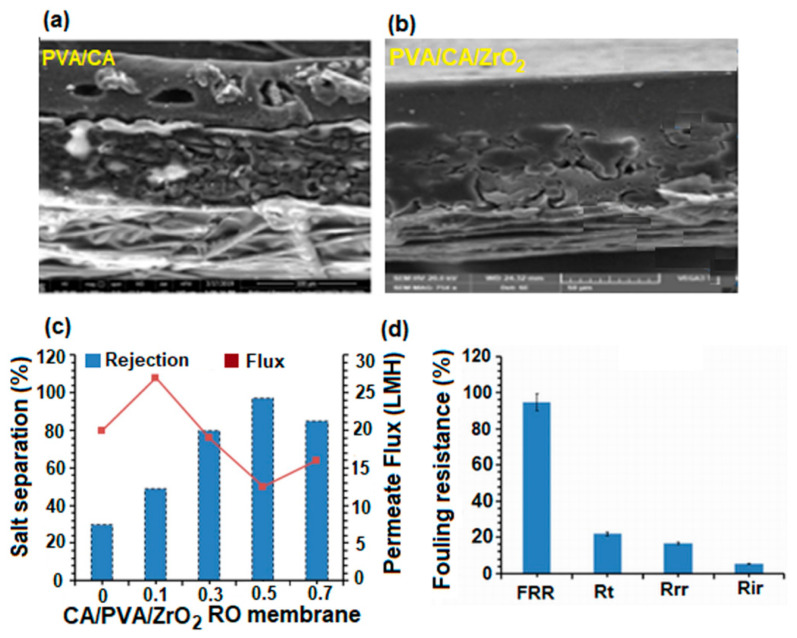
SEM images of the PVA/CA membranes without (**a**) and containing ZrO_2_ NPs (**b**), the separation processes performance (**c**), and the fouling resistance (**d**) of PVA/CA RO membranes. Adapted with permission from [172].

Reverse osmosis is a process for water desalination, which involves a high-pressure domain and uses a semi-permeable membrane with porosity ranging from 0.1 to 1.00 nm. In addition to high hydrophilicity and antifouling properties, RO membranes must exhibit good chemical stability and chlorine resistance [155]. Membranes based on cellulose acetate (CA), PVA, and zirconium dioxide (ZrO_2_) nanoparticles (in different percentages of 0.1, 0.3, 0.5, and 0.7 wt.%), with applicative potential in water desalination were obtained by Alaswad and collaborators [172]. The surface analysis confirmed the obtainment of a dense layer, with specific characteristics of RO membranes. Moreover, tests performed on solutions of 2000 ppm NaCl showed that CA/PVA/0.5 wt.% ZrO_2_ exhibits high water permeability and a salt removal efficiency of 97% (Figure 14). A higher content of ZrO_2_ (0.7 wt.%) led to membrane pores clogging, and implicitly to a decrease in the membrane separation percentage. The chlorine resistance of the membranes was tested for three different pH conditions (pH 4, 7, and 10) without detecting any membrane degradation.

Furthermore, after these tests, the salt retention efficiency was verified, and it was found that the separation percentage was stable even as the chlorine concentration increased. Studies carried out on bovine serum albumin (BSA) solution, used in foulant feeding, showed significant antifouling properties. Lü et al. deposited a layer of polyvinyl alcohol (PVA)/polyquaternium-10 (PQ10) on a polyamide-based RO membrane surface in order to enhance the membrane performance, durability, and antifouling properties [173]. The test results demonstrated that the addition of PQ10 to PVA not only improves the water permeability but also the antifouling properties, as a result of the increasing hydrophilic properties and smoother surface features, but also due to its relatively low surface charge density and pseudo-zwitterionic characteristics. Furthermore, long-term filtration tests demonstrated that although the surface layer has a lower permeability than the uncoated RO membrane, it has a much higher salt removal capacity and a better filtration efficiency of dye wastewater. The resistance to chemicals and chlorine was also tested and it was found that the surface layer of PA-based reverse osmosis membranes improves membrane durability. Therefore, these findings show that the approached technique was a simple method by which the efficiency and durability of the membranes was increased, and which could be promoted as a promising technique for fabricating high-performance RO membranes. Additional research demonstrated a high chemical stability, and good water-softening properties, so that it can be assumed that the obtained membranes are suitable candidates for the intended application. RO membranes based on PA/PVA were obtained by Zhao et al. [174]. PVA was introduced in the aqueous phase (in percentages of 0.001 wt.%, 0.002 wt.%, 0.004 wt.%, and 0.008 wt.%) in order to optimize the membrane surface charge and to prevent membrane fouling. The experimental results showed that the addition of PVA leads to an increase in the water flux to 22.61 L/m^2^h compared to the PA membrane, maintaining a NaCl rejection rate of 97.6%. Tests performed on three types of dyes show that, unexpectedly, negatively charged dye molecules are more easily adsorbed on the negatively charged membrane, which is explained by the layer-by-layer electrostatic attractions between positive and negative charges in the ionic aqueous solution, being easier to remove. The membranes with the highest flux recovery rate for negatively charged dyes were those containing 0.002 wt.% PVA. By comparison, the positively charged dye molecules were more likely to be adsorbed on the membrane’s negative surface and difficult to remove. Through their study, the researchers highlighted the importance of PVA in creating a charged layer on the membrane surface, to prevent membrane fouling in water desalination. Ashrafa and collaborators obtained, characterized, and tested a PVA/CA/PEG composite multilayer membrane (with PVA as a separating layer and CA and PEG as supporting layers) [175]. Tests regarding the desalination capacity of brackish water (13,986 mg/L), groundwater (high saline, 13,986 mg/L), and sea water (excessively salty, 42,847 mg/L) showed a salt rejection of 70%, 63%, and 59%, respectively. After a desalination time of 24 h, the water flux decreased as a result of salt accumulation in the membrane pores. However, a significant reduction in water flux was evident with PEG addition in the membrane. Complementarily, the researchers tested the antimicrobial activity on Gram-positive (*Staphylococcus* sp.) and Gram-negative (*Pseudomonas* sp.) bacteria and found that PVA/CA/PEG membranes with a small pore size (containing PEG of a low molecular mass) show the best antimicrobial activity. The results obtained confirm the possibility of these membranes to be successfully used on an industrial scale in desalination and water purification processes.

### 4.3. PVA-Based Membranes Used in Air Purification

The population growth and intensification of the destructive activities led not only to water pollution but also to air pollution. The main air pollutant is particulate matter (PMx), a mixture of small solid particles and liquid droplets, which can contain both organic and inorganic compounds with toxic or carcinogenic risks. It is classified according to aerodynamic diameter: PM0.1 (ultrafine PM), PM0.3, PM0.5, PM1.0, PM2.5 (fine PM), PM5.0, PM10.0 (coarse PM). The PM indices represent the particle sizes [176,177]. Exposure to these pollutants can cause both respiratory infections and cardiovascular diseases. For this reason, researchers’ attention is focused on finding ways to limit the release of various toxic gases and volatile organic compounds (VOCs) into the air and to minimize as much as possible the negative effects produced by them. The air filtration membranes on the market consist of micro-sized fibers with a low mechanical strength, low thermal stability, and high pressure drops, which do not have a filtration capacity for PM2.5 and PM0.1. Therefore, there is an urgent need to develop filtration devices with improved mechanical properties and low pressure drops, that are reusable and durable, and that can eliminate very fine dust [178,179,180]. Fibrous membranes have attracted attention for their use in air filtration devices due to their high porosity and interconnected pore structure that allows ultrafine filtration [181,182,183].

**Figure 15:**
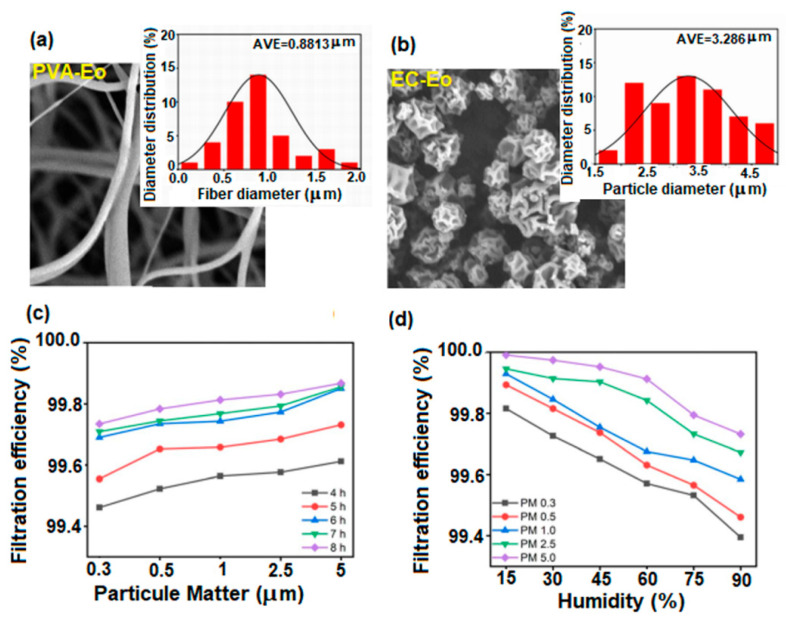
SEM images of the PVA-Eo membrane with the corresponding profile of the fiber diameter distribution (**a**), EC-Eo membrane with a small graph representing the particle diameter distribution (**b**), and filtration efficiency of PMx (**c**,**d**). Adapted with permission from [192].

In recent years, studies focused on the production of water and soil pollution detection devices have been reported [184,185,186,187,188,189,190]. Most often the non-biodegradable polymers used for air filtration devices are polyacrylonitrile (PAN), polyvinylidene fluoride (PVDF), polypropylene (PP), polyamide (PA), and polyvinyl pyrrolidone (PVP). The manufacture of ecological air filtration membranes remains a challenge because the filters obtained from non-biodegradable and non-toxic products cannot ensure durability or reuse but can be successfully used instead of the traditional disposable filters [191]. Polyvinyl alcohol is one of the most reported biodegradable polymers used for the processing of nanofibrous filter membranes. Liu et al. obtained polyvinyl alcohol (PVA)/ethyl cellulose (EC) superhydrophobic bilayer composite membranes [192]. Both polymers (PVA and EC) were mixed with Eugenol (Eo), known as an aromatic, hydrophobic compound, and by its addition the hydrophobic characteristics of the membranes were improved. Thus, the obtained filter consisted of a PVA-Eo electrospun membrane over which a mixture of EC-Eo was electrosprayed. Their filtering capacity was tested on simulated non-oily PMx produced by burning mosquito coils. The results show a high filtration capacity of PMx over 99.6%, at a relative humidity (RH) of 60% (Figure 15). For an RH of 90%, it was found that the filtration capacity decreases, but the obtained results are satisfactory. Therefore, due to their moisture resistance and high filtration capacity, the PVA-Eo/EC-Eo membranes have the potential to be applied as filters or facial masks in air purification. PVA-based filters whose properties have been modified through the addition of GO (in different concentrations of 0 wt.%, 3 wt.%, 6 wt.%, 9 wt.%, 12 wt.%, and 15 wt.%) were obtained by Mohamed and his group [193]. GO presents carboxyl, epoxy, and hydroxyl functional groups that can be greatly exploited in the sense of obtaining membranes with an improved chemical stability but also with good hydrophilicity and antimicrobial properties. The membranes were obtained, characterized, and tested regarding their ability to remove both formaldehyde (FA) (a volatile organic compound—VOC) and sulfur dioxide (SO_2_—a greenhouse gas). Data show that the best performance is achieved by PVA membranes containing 15 wt.% GO with a removal percentage over 90% for FA and 80% for SO_2_.

Zhanga et al. have developed a novel nanofibrous air filter based on electrospun poly(vinyl alcohol) (PVA)/cellulose nanocrystals (CNC), approaching a new, and environmentally friendly way—the heat treatment method—that does not involve the use of other additives. The goal was to create reusable filters with improved water resistance that not requires high energy consumption [84]. The results showed that during electrospinning and heating, the CNCs generate additional nucleation sites promoting the PVA crystallization. Specifically, it was found that for a loading of 20% CNCs and heating at 140 °C for 5 min the crystallinity degree increased from 54.7% to 85.4%. Increasing in samples crystallinity leads to greater moisture stability. The reuse tests were performed for five cycles of washing with water. It was observed that membranes present the possibility of being reused, the filtration efficiency remaining quite high (e.g., PM2.5 were over 95% removed). Considering the non-toxic, biodegradable nature of the components and the ecological method of processing as well as the data obtained, following the membrane evaluations, the PVA/CNCs can be promoted for the industrial scale production of long-lasting filters. Atalie and collaborators have obtained eco-friendly and highly efficient PM0.3 air filter based on natural basalt fiber (BF), cellulose nanocrystal (CNC) fiber and PVA as binder for the fabric [194]. Presence of BF fibers conferred to the nonwoven sandwich-structured mechanical strength, resistance to moisture, heat and fire, while the CNC fibers played an important role in achieving an outstanding filtration efficiency of 99.99% for PM0.3. Advantageous morphological characteristics, thermal stability, mechanical resistance and biodegradability of the obtained membranes make them promising candidates for obtaining the latest generation HEPA filters. Protective masks based on chitosan (CS)/polyvinyl alcohol(PVA) were obtained by Yang et al. [195]. The processing method consisted of the electrospun of CA/PVA mixture and crosslinking of the resulted membrane with glutaraldehyde hydrochloric acid vapor to improve the hydrolysis resistance. The results of the studies showed that the prepared membranes present improved thermal stability, filtration efficiency (>95%), high hydrolysis resistance. In addition, the free aldehyde groups of GA, appearing after crosslinking confer them good antimicrobial properties which is an advantage for the synthesized membranes and target application.

## 5. Sustainable and Environmentally Friendly PVA-Based Food Packaging Material

**Figure 16:**
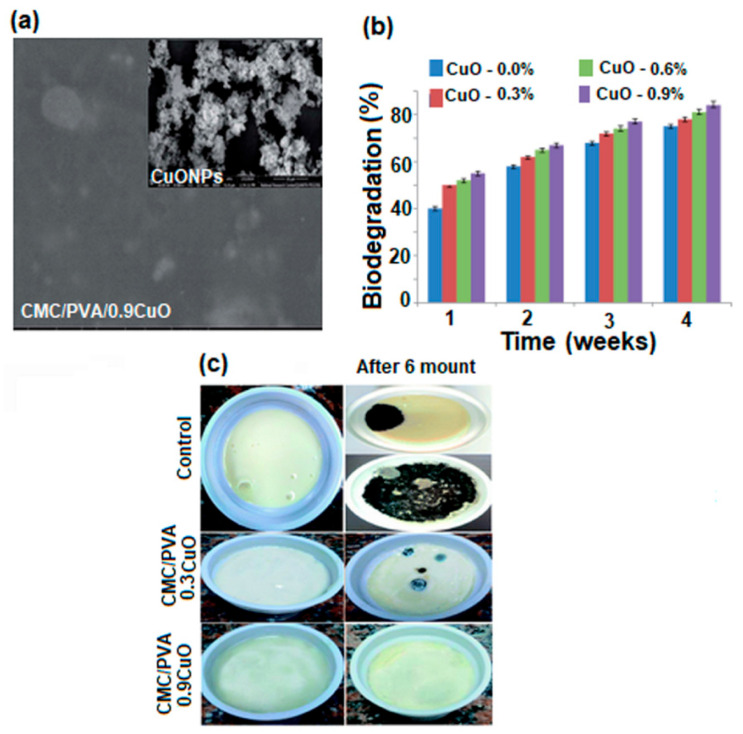
SEM images of CMC/PVA film containing 0.9% CuO with small graph inserted for CuO-NPs (**a**), the biodegradation process of CMC/PVA/CuO-NPs with different content of CuO-NPs (**b**), and the impact of CMC/PVA coatings on cheese after 6 months of cold storage (**c**). Adapted with permission from [211].

The use of single-use plastic food packaging has led to an increase in waste, which poses a great danger to the environment. Thermoplastics such as low-density polyethylene (LDPE), polypropylene (PP), polyvinyl chloride (PVC), and polystyrene (PS), due to their ability to be easily molded and processed, are often used to obtain food packaging [196,197]. These materials have the possibility of being melted and reprocessed, but improperly treated they end up in the environment (water or soil) where they decompose into microplastics, thus representing a risk for animal and human health [198,199,200]. In these circumstances, research is being conducted for the obtainment of non-toxic and biodegradable food packaging by substituting non-biodegradable plastic with natural and renewable resources [201,202]. In addition to polysaccharides [203,204] and proteins [205], biopolyesters such as polyvinyl alcohol and polylactic acid (PLA) have been studied for their potential applications in targeted fields [206]. In recent years, we have witnessed an increased interest on PVA-based materials. Its biodegradable, biocompatible features, water solubility, and low gas permeability make it a highly sought-after polymer for the packaging industry. Its hydrophilic nature allows it to be blended with natural polymers, to create biocomposite films for packaging, with a high water/gas barrier that increases the shelf life of the food. In this context, mixing with inorganic materials, grapefruit seed extract, curcumin, and various essential oils leads to the obtainment of non-toxic, biodegradable PVA films with antibacterial, antioxidant, and UV-protective barrier properties [207,208,209]. Jayakumar et al. obtained, characterized, and tested starch/PVA/zinc oxide (ZnO) nanoparticles composite films as intelligent pH-sensing wraps. They added into the systems nutmeg oil and jamun extract to confer antimicrobial and antioxidant properties to the resulting materials. Data showed that the obtained films have a good mechanical resistance, improved barriers for water and UV radiation, and antimicrobial properties, with potential usage in food packaging applications [210]. In the sense of obtaining sustainable packaging, another evaluated property was the water vapor transmission rate. Thus, in addition to biodegradability, non-toxicity, and antimicrobial and antioxidant properties, an attempt was made to obtain coatings that maintain an optimal humidity so that neither the product nor the packaging deteriorate. Therefore, bio-nanocomposites based on carboxymethyl cellulose (CMC)/polyvinyl alcohol (PVA)/copper oxide (CuO) were formulated and characterized by Youssef and his group [211]. By adding CuO in different quantities (0.3, 0.6, and 0.9 (*w*/*v*)) to the CMC/PVA system, it was observed that the gas and water vapor transmission rate decreased (Figure 16).

Moreover, antimicrobial tests showed an improvement in the inhibitory effect against several pathogenic bacteria and fungi. A significant test consisted of the analysis of cheese covered with such films. After 6 months of cold storage, it was found that the presence of CuO NPs had a major impact on the product preservation by preventing mold and bacterial development. In addition, the coatings prevented the loss of moisture from the product and its drying during storage. The best results were identified for the CMC/PVA mixture containing 0.9% CuO.

In other studies, the grapefruit seed extract and curcumin were used in combination with PVA to obtain films with improved mechanical properties and UV resistance, for their subsequent use as food packaging [212]. As laboratory analyses showed, the antioxidant and antibacterial action of the film were significantly improved with the addition of the two compounds, while the water vapor barrier property slightly decreased, without modifying the thermal stability. Morales et al. obtained, characterized, and tested films from PVA/bio-oils (resulted from base-catalyzed depolymerization of organosolv lignin) [213]. Studies showed that the UV protection capacity and thermal stability of the films increased significantly with the addition of bio-oils, while the mechanical resistance recorded an easy increase. As UV exposure increased, a slight decrease in mechanical resistance of films was observed, still being able to be applied as packaging materials; the process represented the good biodegradation properties of the PVA/bio-oil films. Fasihi et al. added rosemary essential oil (REO) (0.5, 1.5, and 3%) to carboxymethyl cellulose (CMC)/polyvinyl alcohol (PVA) blends emulsified with oleic acid (OL), by the Pickering stabilization method, in order to obtain biodegradable active films with improved antimicrobial and antioxidant properties [214]. The Pickering method involves the use of an emulsion (Pickering emulsions) containing solid particles that stabilize the interface between two immiscible liquids. The process of stabilizing the Pickering emulsion occurs by irreversible absorption of the solid particles at the oil/water interface and generation of a film around the dispersed phase. This acts as a barrier, preventing the contact between the oil droplets and inhibiting the coalescence by steric hindrance. The method is often used in the food industry, biomedicine, and cosmetics industry [215]. In the mentioned study, both PVA and CMC have hydrophilic properties, are not surface-active, what makes them difficult to arrange at the oil and water interface. From this reason an additional step of hydrophobization of the system was imposed. In this context the researchers opted for the use of oleic acid as an emulsifier, which has the capacity to reduce the hydrophilicity of both polymers and facilitates the formation of rigid film around REO. The results revealed a significant increase in antioxidant and antimicrobial activity of the films, especially attributed to the method applied for REO stabilization. The antifungal properties tested both in vitro and on packaged bread slices reveal the antioxidant and antimicrobial activity of REO, suggesting a new way to obtain films with improved properties via stabilization. Composite films based on carboxymethyl cellulose (CMC), poly(vinyl alcohol) (PVA), and ascorbic acid (AA) crosslinked in the presence of glutaraldehyde (GA) were obtained by Hussain and collaborators [216]. Ascorbic acid was used because, unlike metal ions, it represents a safer source that induces antimicrobial properties and can be easily introduced into food foils. In addition, it has antioxidant properties and the ability to regulate the acidity of the packaged products. The polymers were mixed in two ratios, maintaining a fixed proportion of ascorbic acid. The obtained data showed that the CMC/PVA/AA films presented thermal stability, improved mechanical strength, a low water vapor transport rate, and good antimicrobial activity. The best results were obtained for a mixing ratio of 60PVA/40CMC/AA for which the biodegradability rate was also better, making it a good candidate for the food packaging industry.

## 6. Biomedical Applications of PVA-Based Membranes

Polyvinyl alcohol membranes play a significant role in biomedical innovation, finding uses in areas such as drug delivery systems and artificial organ development. By addressing current limitations and integrating interdisciplinary approaches, PVA membranes have the potential to revolutionize healthcare. The beneficial properties of PVA (e.g., biocompatibility, biodegradability, non-carcinogenic nature, capacity for film formation, elevated surface activity, remarkable transparency, etc.) have led to its application in various medical and pharmaceutical fields [115,217,218]. Thus, due to their distinctive characteristics, these membranes are particularly advantageous for a variety of biomedical applications, such as wound dressings, drug delivery systems, tissue-engineering scaffolds, dialysis membranes, and many other uses that benefit from the semi-permeability and superior biocompatibility of PVA (Table 2) [30,219,220,221,222,223]. However, to position PVA as the preferred biomaterial for artificial tissue fabrication, it is vital to thoroughly explore its physiological properties. In this process, analysis of the permeability and diffusion coefficients is particularly important [224]. On the other hand, the degradation rate of PVA can be adjusted by controlling the degree of hydrolysis or crosslinking, making it possible to design membranes that degrade at controlled rates in vivo. This is particularly important in the context of tissue engineering and wound healing, where the scaffold or dressing should be able to support tissue regeneration for a certain period before naturally breaking down and being absorbed by the body [225]. Additionally, enhancing PVA membranes with natural or synthetic polymers, nanoparticles, or other reinforcing materials is particularly beneficial in applications like wound healing and tissue scaffolding, where maintaining structural integrity is vital [30,226,227].

Moreover, PVA is a synthetic macromolecule that has received FDA (Food and Drug Administration) approval for human clinical use and has established a strong reputation in numerous bioengineering applications. The molecular design of biocompatible PVA enables the absorption of protein molecules while ensuring minimal cell adhesion and demonstrating no toxic effects. As a result, its applications include controlled drug delivery, artificial tears, tissue adhesion barriers, hemodialysis, and bone implants, attributed to its beneficial characteristics such as low biotoxicity, high hydrophilicity, and compatibility with renal filtration [290,291,292]. In addition to all this, the presence of hydroxyl groups in PVA enables modifications through the binding of growth factors (GFs) and a range of biomolecules, which can significantly enhance cell adhesion [238,293,294,295].

### 6.1. Drug Delivery Systems

Drug delivery systems play a crucial role in modern medicine due to their significant advantages, including enhanced drug solubility and bioavailability, minimized toxicity to lower the risk of side effects, and the ability to provide successful targeted therapeutic approaches. Moreover, PVA membranes have been widely investigated for their ability to regulate the release of therapeutic agents. Their hydrophilic nature enables controlled swelling, which is critical for sustained drug delivery. While PVA does not biodegrade easily under standard physiological conditions, it is possible to modify or blend it with other polymers to design pH-sensitive systems that allow for controlled drug release, particularly within the gastrointestinal tract. In this context, crosslinked PVA membranes with agents such as glutaraldehyde, formaldehyde, glyoxal, or citric acid have demonstrated prolonged drug release profiles [228,229]. Furthermore, for applications that demand a continuous release of drugs, PVA is used as the core substance for drug incorporation [218,230]. The combination of this polymer with natural polymers has led to its significant use in drug delivery systems. Moreover, PVA electrospun nanofibers, known for their biodegradable and biocompatible properties, are frequently utilized [231,232].

In this regard, PVA/chitosan (CS) nanofibers have garnered significant attention in research owing to their remarkable biocompatibility, biodegradability, and substantial capacity for drug loading. Several studies have investigated the potential of polyvinyl alcohol/chitosan nanofibers in the delivery of diverse drugs, including antibiotics, chemotherapy drugs, and growth factors. The hydrophilic nature of polyvinyl alcohol and chitosan can adversely affect the morphology of PVA, CS, and PVA/CS matrices when they are employed as drug delivery systems. Water absorption can cause swelling, which may lead to a sudden release of the drug. To address this issue, post-treatment is necessary after the electrospinning process to ensure that the nanofibers remain intact during application. The most commonly employed techniques to mitigate the burst release of drugs from nanofibers are chemical and physical crosslinking [233]. For example, Fathollahipour et al. have successfully developed electrospun composite nanofibers of PVA/CS that incorporate gelation nanoparticles, creating a dual drug delivery mechanism [232].

Research efforts have also focused on the utilization of polyvinyl alcohol/chitosan nanofibers for antibiotic delivery systems. According to findings by Kalalinia et al., the incorporation of vancomycin into PVA/CS nanofibers resulted in superior antibiotic activity against *Staphylococcus aureus* infections compared to the administration of the drug in its free state [234]. In the same direction, the research conducted by Abasalta et al. indicates that N-carboxymethyl chitosan-polyvinyl alcohol/poly(3-caprolactone) nanofibers were utilized for the delivery of doxorubicin, demonstrating enhanced therapeutic effectiveness and reduced toxicity when compared to the free form of the drug [235]. Another research study evaluates the potential of polyvinyl alcohol/chitosan nanofibers to serve as a drug delivery system for erythromycin. Hence, this research involved evaluating the in vitro drug release kinetics, biocompatibility, and cellular attachment properties of the nanofibers through in vitro release studies and cell culture assays. The results demonstrated that the PVA/CS nanofibers offered improved in vitro drug release and biocompatibility compared to the free drug formulation [234]. The research study conducted by Madani et al. indicated that polyvinyl alcohol/chitosan nanofibers, when loaded with methotrexate, showed improved in vitro drug release and cellular uptake, suggesting the viability of this system for cancer treatment [236].

On the other hand, according to the research by Cui et al. [233] PVA/CS composite nanofibers containing ampicillin sodium were successfully produced via electrospinning. The crosslinking of these nanofibers was achieved using different concentrations of the crosslinked glutaraldehyde. Analysis through SEM, FTIR, DSC, and DMA confirmed the successful crosslinking reaction, resulting in a well-structured network within the PVA/CS composite nanofibers. The research focused on drug release revealed that the crosslinked network architecture effectively lowered the release rate of ampicillin sodium and mitigated the burst effect when utilizing composite nanofibers.

In the context of developing a drug delivery system for oncology, particularly for breast and liver cancers, a membrane consisting of polyvinyl alcohol and cellulose nanocrystals (PVA/CNCs) has been incorporated with curcumin by Hussein et al. The optimal preparation method for enhancing the hydrogel’s encapsulation capacity was the solution fusion method, utilizing citric acid as a crosslinking agent. Thus, the FTIR spectroscopy results indicated that curcumin and the membrane components are linked through intermolecular hydrogen bonds within the amorphous phase of the PVA/CNC system. Finally, the release profile of curcumin exhibited an initial burst of 41% within the first hour, followed by a sustained release of 70% and 94% over 24 h and 48 h, respectively [296].

**Figure 17:**
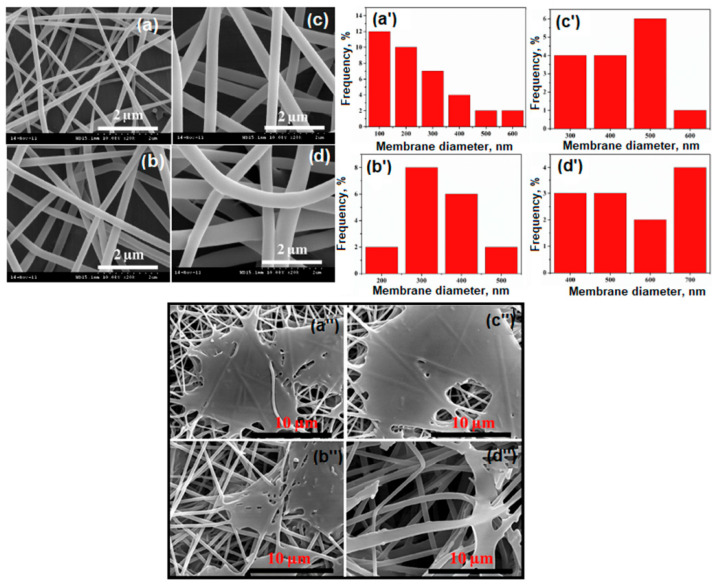
SEM images and the diameter distribution of pure PVA nanofibers obtained from solutions with concentrations of 6 wt.% (**a**,**a’**), 7 wt.% (**b**,**b’**), 8 wt.% (**c**,**c’**), and 9 wt.% (**d**,**d’**). The figure also includes SEM images of the PHA/PVA composite membranes corresponding to the 6 wt.% (**a’’**), 7 wt.% (**b’’**), 8 wt.% (**c’’**), and 9 wt.% (**d’’**) PVA solutions Adapted with permission from [218].

Chen et al. [218] fabricated composite porous membranes consisting of polyhydroxyalkanoate and polyvinyl alcohol (PHA/PVA) by the coupling of electrospinning with spin-coating methods from solutions with concentrations between 6 and 9 wt.%. The outstanding electrospinnability of PVA allowed for the successful production of PVA nanofibers with optimal morphology within a specific concentration range. The porous architecture of the resulting PHA/PVA (as shown by SEM images, Figure 17) composite membrane enhanced material transport, making it suitable for subsequent chemotherapy applications. The results demonstrated that the PHA/PVA membranes have a mesoporous structure, indicating their potential application as carriers for the drug doxorubicin hydrochloride (DOX). In vitro drug release experiments indicated that DOX-loaded PHA/PVA composite membranes exhibited a higher DOX release rate in an acidic environment compared to a neutral environment, attributed to the significantly increased degradation rate of the membranes at pH 4. In addition to investigating drug delivery capabilities, the same research group conducted experiments that revealed the excellent performance of these DOX-loaded membranes for inhibiting the growth of Caco-2 cells. The results showed that the new PHA/PVA porous structure composite matrix has significant potential for use in the chemotherapy of colon cancer. Furthermore, the strong cell adhesion to the membranes suggests that this mesoporous material is also promising for tissue-engineering applications [218].

Studies have demonstrated that PVA possesses excellent biocompatibility and is non-toxic in both in vitro and in vivo environments, being recognized as a standard excipient in numerous pharmaceutical formulations. It is commonly combined with other polymers or materials to improve the composite’s properties and enhance absorption rates.

### 6.2. Wound Dressings

Recent advancements have aimed to improve the properties of PVA membranes by blending them with other polymers or incorporating bioactive compounds to meet specific biomedical needs. These modified membranes have demonstrated effectiveness as wound dressings, providing key benefits such as maintaining a favorable environment for healing, ensuring biocompatibility, and offering antimicrobial protection to prevent infections [237].

Generally, the process of wound healing is intricate and essential for the restoration of the skin’s barrier function. Various diseases can disrupt this process, leading to chronic wounds that pose significant medical challenges. These wounds do not progress through the normal healing stages and are often exacerbated by a pro-inflammatory environment characterized by elevated proteinases, reduced oxygen levels, and bacterial growth [239,240,241]. In this context, a range of advanced medical devices has been created, including wound dressings, wearable monitors, negative pressure therapy systems, and surgical sutures, to improve the conditions of chronic wounds and promote skin tissue regeneration. Many of these devices utilize a diverse selection of materials, ranging from natural and synthetic polymers to bioactive components such as pharmaceuticals, silver, growth factors, stem cells, cytokines, and plant-derived substances. Also, electrospun nanofibers are esteemed as cutting-edge dressing solutions, characterized by their significant porosity and capability to permit air and water permeability. They provide a strong defense against external pathogens and closely replicate the extracellular matrix, making them particularly effective for wound healing and skin regeneration [242].

Due to its excellent moisture absorption capability and biocompatibility, PVA is an ideal material for wound dressings [243,244]. This property is particularly crucial for chronic and diabetic ulcers, where maintaining an optimal moisture balance supports cell migration and facilitates re-epithelialization. Polylactic acid (PLA), in contrast, is valued for its high mechanical strength and biodegradability, making it suitable for scaffold-based dressings that provide structural support and controlled bioactive agent release [297,298]. However, its hydrophobic nature necessitates modifications to improve moisture retention, which can limit its effectiveness in wound environments, requiring sustained hydration. While PLA is advantageous for long-term applications, such as chronic wounds and deep tissue healing [299,300], PVA’s high hydrophilicity and flexibility make it particularly beneficial for acute wounds, burns, and ulcers by promoting moisture retention and patient comfort [301]. PVA membranes effectively maintain a moist wound environment, accelerating healing by minimizing scarring and pain. Although PLA membranes degrade more slowly, making them suitable for prolonged healing processes, PVA offers advantages in terms of cost-effectiveness and ease of large-scale production. Both materials can incorporate bioactive agents, such as antimicrobial compounds or plant extracts, though their release kinetics differ—PLA enables a slower, sustained release, while PVA allows for immediate therapeutic effects [302]. Other biopolymers, including chitosan [303] and collagen [304], are also utilized in wound healing applications. However, PVA distinguishes itself through its optimal combination of moisture retention, flexibility, and manufacturability, making it a more cost-effective option for large-scale wound dressing production.

Coutts et al. [245] explored the effectiveness of co-loading polyvinyl alcohol foam wound dressings with gentian violet and methylene blue for treating chronic wounds in the lower extremities of diabetic patients suffering by bacterial infections. The findings at the end of the assessment period revealed improvements in surface critical colonization and pain scores, particularly in diabetic foot ulcers.

Agarwal et al. [246] investigated the therapeutic potential of curcumin-loaded silk fibroin (SF) nanofibers, blended with varying proportions of PVA and poly(ε-caprolactone) (PCL) for diabetic wound healing. Although PVA has a relatively lower mechanical strength compared to poly(ε-caprolactone) [247], various concentrations of PVA are employed alongside PCL to address different types of wound care. The study demonstrated that the nanofibers possessed the necessary physicochemical properties for dermal application. SEM analysis revealed the presence of smooth, fine fibers arranged in a nanoscale network structure. Additionally, the nanofibers exhibited suitable mechanical strength and biodegradability. The researchers reported significant wound-healing effects in diabetic mice, with curcumin-loaded SF/PCL and SF/PVA nanofibers achieving wound area reductions of 99% and 96.54%, respectively, after 14 days of treatment. Also, nanofibers composed of PVA/CS and infused with the silk protein sericin were produced using the electrospinning method. These nanofibers, containing a minimal amount of sericin, showed improved cell proliferation relative to PVA/chitosan nanofibers without sericin. Furthermore, studies conducted on animals validated their potential for wound healing [248].

Composite membranes made from PVA-biopolymers, which include healing agents like Aloe vera, PEG, and sterculia/arabic gums, along with antibiotics such as ampicillin or gentamicin, are proposed as typical dressings for managing acute and chronic wounds. In this context, Uslu et al. developed PVA-PVP-PEG nanofiber mats. They incorporated hydroxypropyl methylcellulose (HPMC) into the nanofibers due to its excellent water retention characteristics, along with Aloe vera which acts as a healing agent. The integration of Aloe vera significantly modified the nanofibers’ morphology, resulting in a porous structure that promotes effective vapor and oxygen permeability. Moreover, its notable therapeutic properties against microbial growth were assessed, confirming its role as an effective agent in enhancing the healing process [249].

The literature data suggest that gamma radiation is an effective method for crosslinking PVA/chitosan dressing membranes. The findings indicate that increasing the PVA content or irradiation dose enhances mechanical properties and resistance to microbial penetration, attributed to the formation of a strong crosslinked structure. Instead, higher chitosan content improves the membranes’ swelling capacity and antimicrobial properties [250].

In a recent investigation by Najafi et al. [251], alginate/PVA nanofibrous mats were developed incorporating cardamom extract for antibacterial wound dressing applications. The results of the antibacterial testing indicated that the nanofibrous web loaded with cardamom extract exhibited remarkable antibacterial properties, with effectiveness rates of around 99% against Gram-positive bacteria and 97% against Gram-negative bacteria.

Furthermore, antimicrobial PVA membranes are increasingly used in the treatment of infected wounds. The incorporation of antimicrobial agents, such as silver nanoparticles, into PVA membranes imparts antibacterial properties, reducing the risk of infection while supporting wound healing [252]. In this context, Nematollahi and collaborators investigated the development of advanced wound dressings with enhanced antibacterial properties for the treatment of second-degree burn wounds [253]. Their study focused on the synthesis of nanofibers by incorporating silver nanoparticles coated with polyhexamethylene biguanidine (Ag/PHMBG) into chitosan–thiourea (CST) matrices. Due to the electrospinning challenges associated with chitosan’s cationic nature, PVA was used as a blending agent to enhance its processability. To improve mechanical properties and dimensional stability, thermal annealing was employed as an alternative to conventional chemical crosslinking. In vitro studies demonstrated significant antimicrobial efficacy, with a 95% reduction in bacterial growth (both Gram-positive and Gram-negative) within three days, without cytotoxic effects on normal fibroblast cells. Additionally, in vivo studies in animal models revealed accelerated wound healing and tissue regeneration. These findings suggest that the developed nanofibers are promising materials for wound healing applications, offering strong antibacterial properties and biocompatibility.

**Figure 18:**
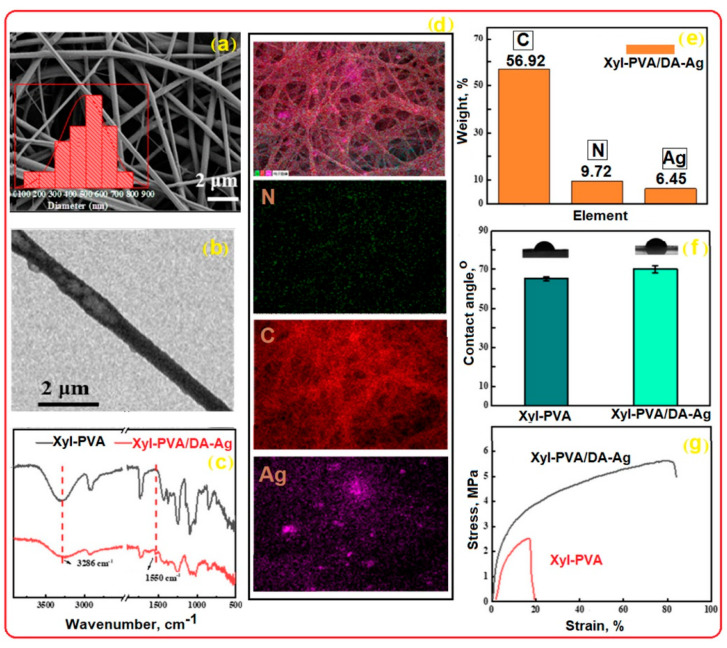
The schematic illustration includes SEM images and the distribution of fiber diameters (**a**), TEM images (**b**), FTIR spectra (**c**), EDS mapping (**d**), and the elemental composition of Xyl-PVA/DA-Ag nanofiber mats as determined by EDS (**e**). Additionally, it presents the contact angles (**f**) and tensile strength (**g**) of both Xyl-PVA and Xyl-PVA/DA-Ag nanofiber mats. Adapted with permission from [238].

Yang et al. [238] conducted a study highlighting the impact of nanofiber-based wound dressings enhanced with silver nanoparticles (AgNPs) and dopamine (DA) on wound treatment. By employing coaxial electrospinning, they successfully incorporated small-molecule sugar alcohols (SAs), such as xylitol (Xyl), sorbitol (Sor), and erythritol (Ery), into a PVA nanofiber matrix enriched with AgNPs and DA. These coaxial electrospun nanofiber dressings were designed to offer self-adhesive properties, effective elimination of reactive oxygen species (ROS), and a superior antimicrobial performance. Comprehensive characterization of the Xyl-PVA/DA-Ag nanofiber mats was performed using SEM, TEM, FTIR, EDS, contact angle analysis, and tensile strength testing (Figure 18).

TEM confirmed the formation of a core–shell structure, while EDS analysis revealed substantial DA and Ag content, attributed to DA self-polymerization and silver chelation. The composite fiber membranes incorporating DA and AgNPs exhibited excellent hydrophilicity with only slight changes in the water contact angle, while their mechanical strength improved due to the strong adhesion of DA, which facilitated multiple hydrogen bond formations. The Xyl-PVA/DA-Ag nanofiber mats demonstrated outstanding adhesion in humid environments, along with moisture absorption and cooling properties, making them highly suitable for exudative wound treatment. The presence of xylitol and dopamine contributed significantly to ROS scavenging and enhanced antioxidant activity. As a result, these composite nanofibers exhibited self-adhesive behavior and remarkable antimicrobial efficacy, achieving an antibacterial rate exceeding 99%. This study underscores the potential of Xyl-PVA/DA-Ag nanofiber dressings as advanced materials for wound healing, combining structural integrity, biocompatibility, and therapeutic functionality.

Based on this approach, several studies have demonstrated the significant potential of nanofiber-based wound dressings incorporating chitosan, PVA, and zinc oxide (ZnO) for diabetic wound healing. Ahmed et al. [254] reported that electrospun chitosan/PVA/ZnO nanofibrous mats exhibited notable antibacterial and antioxidant properties, which are crucial for promoting wound healing in diabetic patients. These characteristics highlight the effectiveness of ZnO-containing nanofibers in preventing infections and mitigating oxidative stress, both of which are major challenges in chronic wound management. Furthermore, membranes composed of PVA-chitosan-ZnO demonstrated strong antibacterial activity against *Staphylococcus aureus*, attributed to the presence of ZnO nanoparticles. In these formulations, glycerin or Tween-80 acted as a plasticizer, improving the flexibility and usability of the membranes [255]. Similarly, Shalumon et al. [256] found that PVA–alginate–ZnO nanofiber mats exhibited potent antibacterial effects against *Escherichia coli* and *S. aureus*, even with low ZnO concentrations. Beyond their antimicrobial efficacy, the incorporation of ZnO nanoparticles has been instrumental in enhancing the mechanical and thermal properties of membranes and nanofiber mats. These improvements contribute to the overall stability and durability of the dressings, making them more effective in providing a protective and bioactive environment for wound healing. This research underscores the promise of ZnO-integrated nanofibers as advanced wound care materials, capable of accelerating tissue regeneration while preventing infection.

**Figure 19:**
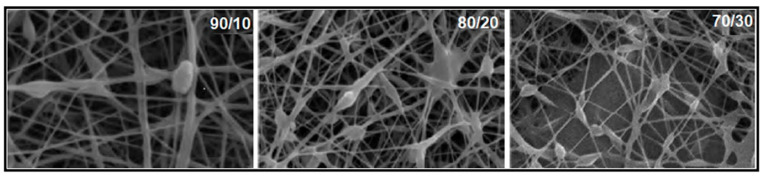
SEM images of electrospun nanofibers obtained from 4% calcium alginate and 7% PVA solutions in different volume ratios Adapted with permission from [257].

**Figure 20:**
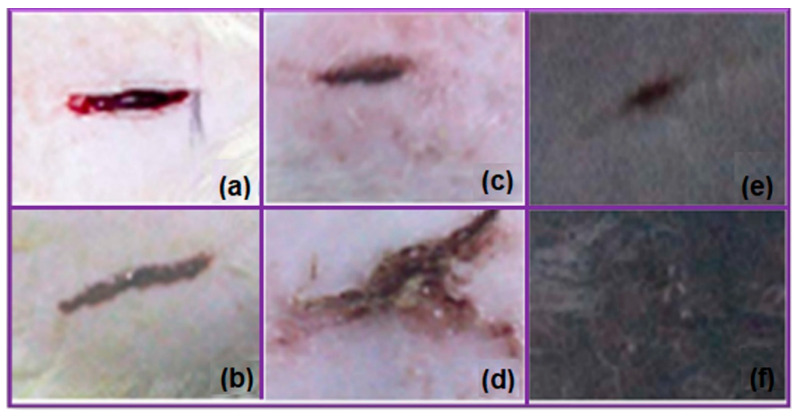
Schematic representation showing the examination of PVA–alginate nanofiber matrix in vivo studies on wound healing: (**a**,**c**,**e**) control wounds, and (**b**,**d**,**f**) test wounds Adapted with permission from [257].

Tarun et al. [257] introduced an innovative approach to developing PVA/calcium alginate composite nanofiber networks for wound healing. Using the electrospinning technique, they blended PVA with calcium alginate to improve the spinnability of the solution and enhance the mechanical properties of the resulting fibers. The study demonstrated that increasing the calcium alginate content (90/10, 80/20, and 70/30 wt./wt.) resulted in favorable morphological characteristics and optimal water vapor transmission properties, creating an optimal environment to accelerate wound healing (Figure 19). In vivo studies using the nanofibrous membrane with the highest alginate content (70/30 PVA/calcium alginate) confirmed its effectiveness in promoting wound healing in female rats. As illustrated in Figure 20c, after five days of evaluation, the control wound remained open, measuring 0.7 cm, whereas the test wound covered with the nanofiber matrix had reduced to 0.5 cm (Figure 20d). By day eight (Figure 20e), the control wound showed some epithelial cell growth but remained open. In contrast, the test wound (Figure 20f) exhibited significant epithelial cell proliferation, resulting in complete wound closure. These findings highlight the potential of PVA/calcium alginate composite nanofibers as effective wound dressings, offering structural integrity, moisture balance, and an environment that accelerates tissue regeneration and healing.

Recent research by Suhail et al. [305] has led to the development of self-healing membranes by incorporating tannic acid (TA) into PVA matrices, offering significant advancements in skin bio-applications. These PVA-TA membranes exhibit remarkable mechanical strength and self-repair capabilities, making them well suited for dynamic skin environments. The study reported a tensile strength of 8.8 MPa before healing and 7.6 MPa after healing, corresponding to an impressive healing efficiency of 88%. Furthermore, the membranes demonstrated exceptional durability, retaining flexibility even after 3000 bending cycles. Notably, no significant variations were observed in the water vapor transmission rate or oxygen transmission rate, ensuring their stability and effectiveness in maintaining a favorable microenvironment for wound healing. These findings highlight the potential of PVA-TA membranes as innovative materials for advanced skin bioengineering, providing both structural resilience and enhanced self-repair properties essential for next-generation wound care and regenerative medicine.

### 6.3. Tissue Engineering and Regenerative Medicine

**Figure 21:**
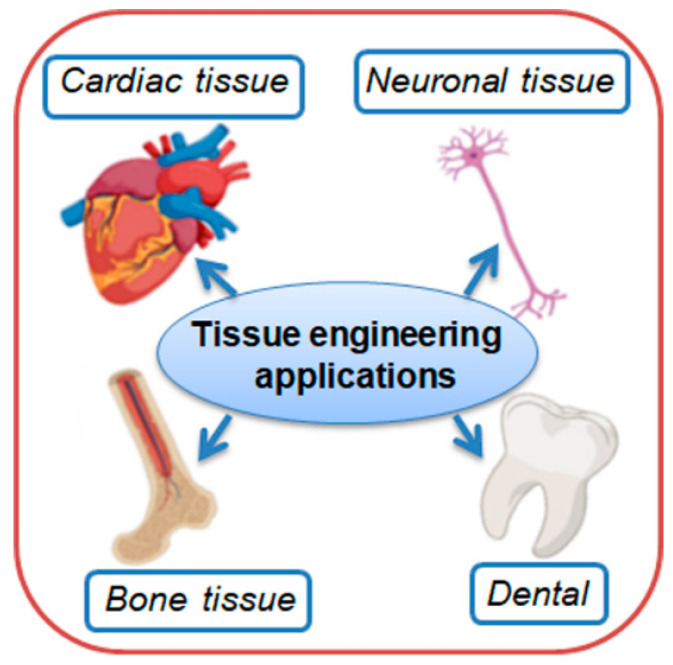
A schematic illustration of applications in tissue engineering. Adapted with permission from [30].

Tissue engineering is another area in which PVA membranes have shown considerable promise. The ability of PVA to form porous, biocompatible scaffolds makes it an excellent candidate for the obtainment of artificial tissues (e.g., bone and skin) and organs. In the field of tissue engineering, PVA membranes serve as scaffolds that facilitate cell adhesion, proliferation, and differentiation (Figure 21) [30,305].

**Figure 22:**
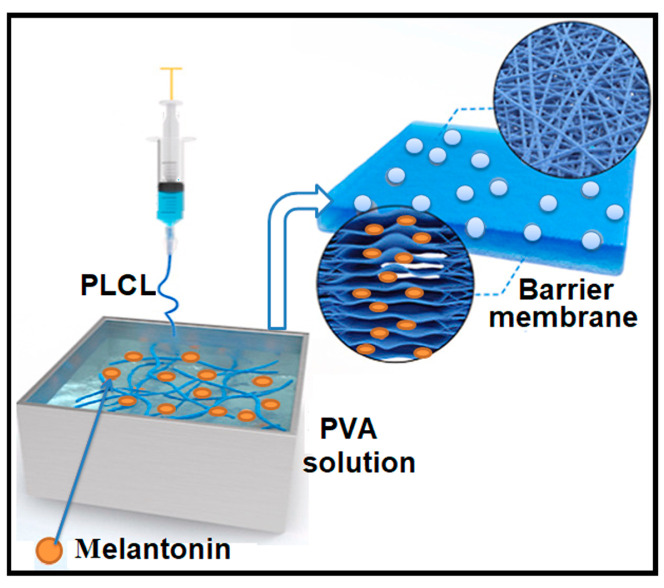
Schematic representation showing modified electrospinning method incorporating PLCL nanofibers, PVA aerogel, and melatonin. Adapted with permission from [260].

The research by Zhang et al. indicated that a flexible conductive polymer sheet utilizing carbon black (CB) and PVA can be successfully used in identifying small strains and tracking humidity levels, with significant implications for biomedical use. Their investigation demonstrated an excellent sensing ability and stability across various humidity levels, from a relative humidity of 10% to 70% [259]. Also, a functional membrane was developed using a modified electrospinning method, incorporating poly(L-lactide-co-caprolactone) (PLCL) nanofibers, PVA, and melatonin (Figure 22).

This innovative membrane presents porosity and hydrophilicity provided by PVA, while the PLCL nanofibers contribute to its mechanical properties, making it suitable for suturing applications. To evaluate its effectiveness and the potential mechanisms for preventing pericardial adhesion, both in vitro and in vivo studies were conducted. The results demonstrate the significant potential of the membrane for practical anti-adhesion applications [260].

**Figure 23:**
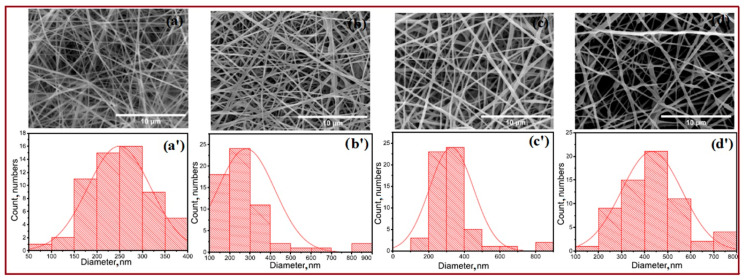
SEM images (**a**–**d**) together with respective histograms that depict the diameter distribution (**a’**–**d’**) for nanofibrous scaffolds. The nanofiber categories represented include PVA (**a**,**a’**), PVA90/EX10 (**b**,**b’**), PVA80/EX20 (**c**,**c’**), and PVA70/EX30 (**d**,**d’**). Adapted with permission from [220].

In their research, Asran et al. obtained PVA/PB (polyhydroxybutyrate) electrospun membranes aimed at serving as skin substitutes. In studies conducted on the human keratinocyte cell line (HCT) and dermal fibroblasts, maximum adhesion and proliferation were observed on pure PHB. Moreover, the introduction of 5 wt.% PVA to polyhydroxybutyrate inhibited the growth of human keratinocyte cell line cells, although fibroblast growth was not affected. In contrast, the PVA/PB (50/50 wt./wt.) fibers enhanced the adhesion and proliferation of HCT cells, while inhibiting fibroblast growth [261]. Another research study focuses on the incorporation of flaxseed extract (EX), rich in polyphenolic compounds, into polyvinyl alcohol nanofibers [220]. To optimize the cellular responses connected to the nanofiber scaffolds, it was imperative to carefully control key parameters like the fiber diameter, wettability, tensile strength, swelling ratio, and hydrolytic degradation. Thus, the SEM results showed that nanofibrous scaffolds enriched with flaxseed extract have a uniform structure without beads and a variety of fiber diameters, which are crucial for successful bone tissue regeneration (Figure 23). In addition, biological testing involving MG-63 osteoblast-like cells indicated a significant enhancement in both cell proliferation and migration, supported by MTT and cell migration assays, when utilizing PVA nanofibers infused with flaxseed.

By combining PVA with collagen (Coll) or chitosan, membranes can be achieved with enhanced biocompatibility and functional properties that are more effective in tissue-engineering applications. In this context, the PVA-CS scaffold serves as an effective platform for tissue regeneration, whether utilized independently or in conjunction with other polymers and cellular elements [262,263]. Castro et al. formulated four novel composite films using PVA-CS and tea tree (Melaleuca alternifolia) essential oil, which were subsequently assessed through subdermal implantation in Wistar rats. The results showed that the films exhibited a biocompatibility similar to that of porcine collagen, especially those with an increased content of tea tree essential oil [264]. Bilayered electrospun membranes for skin tissue engineering and wound dressings were obtained by combining the PVA, CS, Coll, and tamarind seed polysaccharide (Tsp), subsequently crosslinked with quercetin [265]. In order to achieve a prolonged drug release from the membrane, a novel stable PVA fibrous mat with a crosslinked two-layer structure was designed. The top layer consisted of PVA/CS/Tsp and quercetin, while the bottom one comprised PVA/CS/Coll. The results indicated the successful separation of fibrous bilayers, confirming the bilayered scaffold’s uniform porosity, which is advantageous for tissue-engineering purposes. In vitro biological studies showed an encouraging cell viability of fibroblast cells, with enhanced proliferation and adhesion on the bilayer material, indicating its potential for targeted applications in tissue engineering.

**Figure 24:**
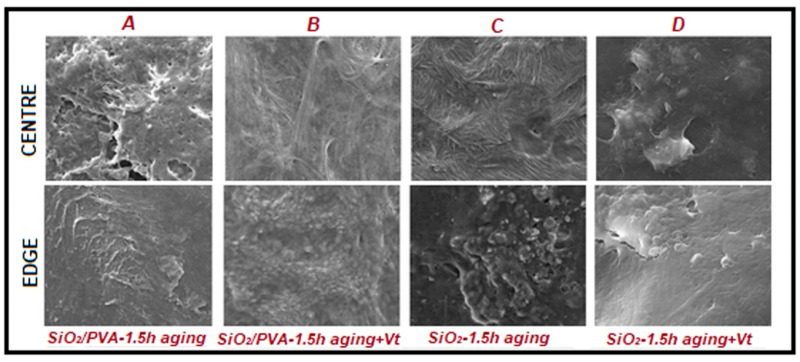
SEM images depicting the bone samples collected four weeks following the surgery. Adapted with permission from [266].

An advanced strategy for developing high-performance PVA membranes involves coupling electrospinning with 3D-printing techniques to fabricate fibrous scaffolds incorporating silicon dioxide (SiO_2_). This innovative approach was explored for its potential in bone tissue engineering, using in vivo studies conducted on an animal model to assess the membranes’ bioactivity and regenerative potential [266]. The results revealed that calcined silica fibrous scaffolds demonstrated exceptional mineralization and significantly enhanced bone regeneration. Notably, the incorporation of vitronectin (Vt) into the calcined networks further accelerated mineralization and promoted osteogenesis by facilitating cell adhesion to the fibrous matrix. The SEM images of bone samples taken four weeks post-surgery confirmed a widespread distribution of osteoblast populations across all experimental groups (A, B, C, and D), with the highest cell densities observed in vitronectin-containing scaffolds, underscoring its pivotal role in cell adhesion and differentiation (Figure 24). The only instance where the fibrous matrix cannot be recognized is found within the SiO_2_-1.5h aging + Vt group.

Furthermore, in vivo evaluations reinforced the scaffolds’ effectiveness as bioactive materials, demonstrating their ability to enhance osteointegration and bone regeneration. Their superior physicochemical properties, combined with their capacity to promote mineralization and cellular activity, establish PVA/SiO_2_ fibrous scaffolds, particularly those reinforced with vitronectin, as highly promising candidates for advanced bone tissue-engineering applications [266]. The integration of titanium dioxide (TiO_2_) into PVA membranes has been explored to enhance bioactivity, particularly for applications in bone tissue engineering. Nguyen et al. [267] investigated the ability of TiO_2_-infused membranes to promote hydroxyapatite (HA) formation when immersed in simulated body fluid, a crucial factor for bone regeneration. Their findings revealed that membranes containing rutile-phase TiO_2_ exhibited a greater degree of HA mineralization compared to those with anatase-phase TiO_2_. Moreover, prolonged immersion significantly influenced HA formation, with membranes exposed for three weeks demonstrating substantially higher levels of HA deposition. This highlights the critical role of both TiO_2_ phase composition and immersion duration in optimizing bioactivity. Additionally, in vitro studies confirmed the biocompatibility of HA/PVA/TiO_2_ membranes, as evidenced by high cell viability, reinforcing their potential use in biomedical applications. These findings suggest that TiO_2_-enhanced PVA membranes hold promise as bioactive materials for bone tissue engineering, supporting cell adhesion, mineralization, and overall tissue regeneration.

The development of PVA/CS nanofibers reinforced with carbon nanotubes, either independently or in combination with bioactive glass particles, was aimed at improving the porosity, structural integrity, and biocompatibility of PVA-based scaffolds for neural tissue engineering [268,269]. These modifications were designed to create an optimized microenvironment for nerve regeneration. In this direction, in vitro evaluations demonstrated that the resulting scaffolds exhibited enhanced mechanical properties, facilitating improved cell adhesion and proliferation. Furthermore, in vivo studies confirmed the efficacy of guidance conduits composed of PVA integrated with functionalized multi-walled carbon nanotubes (MWCNTs) for peripheral nerve regeneration, highlighting their potential as advanced biomaterials for neural repair applications [270]. Additional findings revealed that electrically conductive tube-guides, particularly those composed of PVA-CNTs, significantly enhance nerve regeneration in rat sciatic nerve models [271]. These conductive scaffolds not only promote axonal regrowth but also mitigate neurogenic atrophy in muscles affected by nerve damage, a common consequence of severe injuries and extended recovery periods. As a result, they facilitate improved functional recovery and myelination of regenerated nerve fibers while preventing inflammatory responses, highlighting their potential as advanced biomaterials for neural repair.

Postoperative adhesions are common complications following surgical interventions, particularly in the abdominal region, leading to chronic pain, infertility, and impaired organ function. PVA membranes have been identified as promising solutions to mitigate adhesion formation by acting as temporary physical barriers that separate tissues during the healing/recovery process [272]. In this direction, Bae et al. [273] developed PVA/gelatin (PVA/Gel) composite membranes with varying compositions (10/90, 30/70, and 50/50 wt./wt.) and investigated their material properties and biocompatibility for adhesion prevention. The results revealed that membranes with a higher gelatin content exhibited superior hydrophilicity and enhanced cell viability. Both in vitro and in vivo evaluations demonstrated that the PVA/Gel (10/90 wt./wt.) membrane was particularly effective in preventing adhesions, underscoring its potential for biomedical applications.

Furthermore, another approach involved the application of PVA coatings on electrospun poly(lactide-co-glycolide) (PLGA) scaffolds via dip-coating in aqueous solutions containing 3 wt.%, 6 wt.%, and 9 wt.% PVA. This dip-coating technique was selected for its simplicity and efficiency in uniformly coating substrates of varying geometries. The study systematically analyzed the morphological, physicochemical, cytotoxic, and anti-adhesive properties of both uncoated and PVA-coated PLGA scaffolds. The results confirmed that PVA-coated PLGA membranes exhibited superior biocompatibility, flexibility, and anti-adhesive properties, making them promising candidates for preventing intra-abdominal adhesions in biomedical applications [274].

As a result, PVA demonstrates significant potential for the development of scaffolds with tailored mechanical properties, making them highly suitable for a wide range of tissue-engineering applications. The ability to modify PVA’s characteristics and production methods and incorporate various additives enables the creation of scaffolds that closely mimic the mechanical strength of natural tissues. With continuous advancements in PVA scaffold refinement and the exploration of innovative composite materials, PVA-based scaffolds are poised to become a leading choice in tissue engineering.

### 6.4. Hemodialysis and Artificial Organs

**Figure 25:**
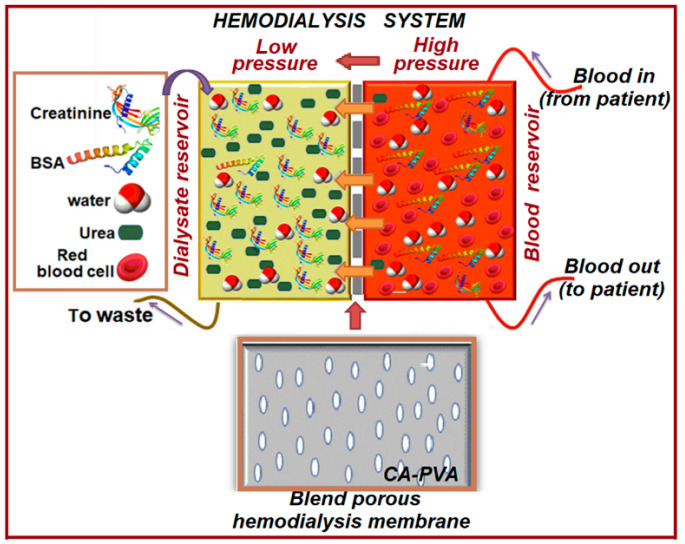
Schematic illustration of hemodialysis system. Adapted with permission from [276].

The use of PVA membranes in hemodialysis represents another important biomedical application. As a therapeutic approach for end-stage renal disease, hemodialysis employs semi-permeable membranes to differentiate between proteins and uremic toxins based on their molecular weights. PVA-based membranes are ideal for this application because they provide high permeability and are biocompatible with human blood, reducing the risk of adverse reactions [275]. To obtain a high-quality hemodialysis membrane, in addition to essential attributes such as highly permeability and selectivity, various properties are useful, including strong resistance to protein fouling, reduced thrombotic potential, and effective cytocompatibility. Furthermore, the surface morphology and chemistry of the membrane can be refined by adjusting the proportions of the polymer, solvent, and additives in the prepared solution. Also, the physicochemical properties (e.g., pore size, mechanical strength, hydrophilicity, and hemocompatibility) can be improved by using blends or replacing membranes with alternative materials [276,277,278]. Therefore, Azhar et al. focus on improving the filtration efficiency and biocompatibility of cellulose acetate (CA) hemodialysis membranes by incorporating PVA and polyethylene glycol (PEG) as additives. The biocompatibility of CA-PVA membranes was evaluated through various tests, including platelet adherence, hemolysis proportion, thrombus evolution, and plasma recalcification time (Figure 25). In addition, the membrane displayed a uniform porous configuration, and PVA effectively increased the selectivity of the membranes by reducing the pore size. The results indicated that the platelet adhesion and hemolysis ratio for the cast membranes were lower than those of the pure CA membrane. Furthermore, the membranes demonstrated increased clotting times and reduced thrombus formation on their surfaces, indicating their biocompatibility. Consequently, CA-PVA hemodialysis membranes were shown to be more efficient than the traditional hemodialysis membranes reported in the literature, highlighting their potential as high-performance biocompatible membranes [276].

In this direction, Yu et al. created a thin-film nanofibrous composite membrane, characterized by high permeability, having an ultrathin hydrophilic active layer composed of chemically crosslinked PVA and a nanofibrous support layer of electrospun polyacrylonitrile (PAN). Thus, the improvement of the active PVA separation layer facilitated the creation of an ultrathin active separation layer distinguished by advantageous diffusive transport properties. This ultrathin PVA layer exhibited significant selectivity, characterized by a small pore-size distribution, which effectively eliminated uremic toxins while preserving proteins. Also, the nanofibrous support layer provided high permeability, featuring a greatly interconnected pore structure, which facilitated the transfer of toxins during hemodialysis [279].

In their study, Song et al. developed a heparin-like membrane by applying carboxymethyl chitosan and PVA nanoparticles to modified bacterial cellulose sulfate membranes using electrospinning techniques. Thus, the heparin-like membrane shows similarities to heparin due to the presence of -SO_3_, COO-, and -OH groups on their surfaces. Furthermore, the heparin-like membrane demonstrated a higher hydrophilicity compared to the bacterial cellulose sulfate membrane, potentially contributing to its anticoagulant properties [83]. Furthermore, Ding et al. [280] developed a PVA/PAN-U-60 thin-film nanofiber composite membrane. This advanced membrane was combined with PVA hydrogeland enhanced with UiO-66-(COOH)_2_ nanoparticles, serving as a separation coating. The top layer consisted of an ultrathin PVA hydrogel coating that facilitated the rapid passage of toxins through the cortex to the dialysate while effectively preventing the leakage of significant proteins. Thus, the methodology involved the creation of a PAN nanofibrous membrane enriched with UiO-66-(COOH)_2_ nanoparticles by colloidal electrospinning, which serves as the substrate for the composite membrane used in hemodialysis for creatinine removal. This membrane eliminated a significant amount of contaminants from the blood while ensuring a high level of protein retention. Consequently, the inclusion of a porous substrate in the hemodialysis composite membrane, which possesses adsorption capabilities, could greatly reduce the volume of dialysate needed. The results of this research could lead to significant progress in lightweight design innovations and support the creation of portable artificial kidney systems [280]. Another research study aimed to create alginate-based biopolymers with superior physical and chemical attributes obtained by esterification with PVA, designed to be used as a biocompatible membrane in the context of hemodialysis. Therefore, PVA was chosen as a polymer modifier due to its exceptional mechanical strength, biocompatibility, and non-toxic property. Following modification with PVA, the hydrophobicity of the membrane was significantly improved, which is reflected in the increased water contact angle. Low protein attachment and platelet adhesion showed that PVA–alginate membranes were more hemocompatible than native alginate membranes. Therefore, the PVA–alginate ester could serve as a potential replacement for cellulose acetate in the fabrication of hemodialysis membranes [281].

### 6.5. Other Uses of PVA Membranes in the Biomedical Field

PVA finds extensive use in multiple applications, frequently alongside various polymers, particularly in the context of periodontal treatment [282], ophthalmic, orthopedic, cancer therapy, immunotherapy, gene therapy, and cosmetics [264,283,284,285,286].

In periodontitis treatment, the localized delivery of medications or antimicrobial agents is a suitable approach when an infection is highly concentrated and shows limited response to mechanical cleaning or systemic antibiotics [287]. The research conducted by Santos et al. involved the development of core-sheath nanofibers via coaxial electrospinning, using chitosan (of low (CL) and high (CH) molecular weight) as the shell material and PVA containing tetracycline hydrochloride (TH) as the core component. The obtained membrane was crosslinked with genipin (G) and its subsequent evaluation in terms of physical–chemical–biological properties demonstrated a decrease in hydrophilicity, an increase in mechanical strength, and an improvement in biocompatibility. Additionally, the crosslinked nanofiber membrane showed considerable stability in water, favoring the sustained release of pharmaceuticals. In vitro antimicrobial analysis revealed that only the crosslinked nonwovens containing TH, especially the one obtained from chitosan of high molecular weight, G(CH/PVA-TH), exhibited significant efficacy against bacterial strains related to periodontal disease. Furthermore, these nonwovens were found to be non-cytotoxic to fibroblast cells, suggesting their viability as an innovative drug delivery platform for periodontitis cure [288]. The investigation carried out by Joshi et al. focused on the synthesis of nanofibers that exhibit both controlled-release functionality and strong adhesion properties by blending hydroxyapatite with PVA, thereby establishing an effective drug delivery system for periodontitis treatment. PVA serves as an effective penetration amplifier, facilitating the absorption of the encapsulated drug and thereby enhancing its pharmacological efficacy. Both in vitro and in vivo investigations revealed the broad-spectrum antimicrobial effectiveness, highlighting the potential of biomimetic nano-hydroxyapatite for addressing periodontal defects [289].

Recently, scaffolds processed by tissue engineering have been recognized as a potential therapeutic strategy for the repair and regeneration of tissue defects related to periodontal disease. In this regard, a recent investigation revealed that PVA/CS has the potential to serve as a remarkable flexible membrane used in periodontal regeneration, effectively inhibiting fibroblast infiltration into the wound and proving beneficial in periodontal regeneration procedures. Therefore, PVA/CS films with different tensile strengths were developed and assessed for their cell toxicity, biodegradation properties, and swelling ratios in the context of periodontal regeneration surgery. These films are characterized by good biodegradation capabilities, allowing them to maintain their functional properties throughout the critical phase of periodontal regeneration (8 weeks). It was also observed that the hydrogel films containing PVA exhibit a bilayered architecture, comprising both dense and porous structures. In vitro cytotoxicity evaluations showed that the PVA/CS hydrogel exhibits remarkable biocompatibility, especially with the human osteosarcoma cell line (MG63 cells), which is relevant for the evaluation of bone regeneration. As a result, these versatile PVA/CS films are suitable for periodontal regeneration procedures and potential clinical applications [282]. The research realized by Dang et al. [284] involved the preparation of chitosan (CS)/PVA/graphene oxide (GO)/astaxanthin (ASTA) nanofiber membranes aimed at modulating inflammatory responses and promoting osteogenic induction in vitro. The findings indicate that the electrospun CS/PVA/GO/ASTA nanofiber membranes exhibited favorable micro-morphology, robust mechanical properties, and non-cytotoxicity. Furthermore, the results suggest that these nanofiber membranes could facilitate a reduction in inflammation and encourage the differentiation of bone marrow mesenchymal stem cells into osteoblasts [284]. In addition to PVA, other viable options suitable in tissue-engineering applications include PLA, polycaprolactone (PCL), polyglycolic acid (PGA), poly(lactic-co-glycolic acid) (PLGA), chitosan, hyaluronic acid (HA), Coll, and polyethylene glycol (PEG). These polymers facilitate innovative biomedical applications providing essential properties [306,307,308,309]. PLA is favored for its mechanical strength and biodegradability, making it suitable for tissue-engineering applications, especially in orthopedic diseases. From this reason, blends from PLA and PVA were selected, taking into account their specific properties and particular needs from the medical field [310,311,312,313]. In this sense, Sharma et al. [309] focused on the development of polylactic acid (PLA)/poly(ε-caprolactone) (PCL) electrospun mats (EMs) coated with polyvinyl alcohol (PVA) impregnated with curcumin-β-cyclodextrin inclusion-complex (IC). The new complex structure was evaluated in order to obtain fibrous membranes with improved antibacterial activity that facilitate cell attachment and regeneration. The results of the studies showed that a content of IC over 60 wt.% in the PVA matrix exhibits a substantial increase in the antibacterial efficacy, while tests on fibroblast-like cells (L929) revealed an excellent cell viability of 135% for a loading with 20 wt.% IC. Therefore, the inclusion of curcumin-β-cyclodextrin complexes ensures an enhancement in antibacterial activity, while PVA confers mechanical strength and flexibility and helps in cell attachment, ensuring a uniform distribution of curcumin-β-cyclodextrin complexes. In addition to these advantages, PLA contributes to increasing the membrane biocompatibility, biodegradability, and mechanical integrity, supporting the controlled release of the curcumin complex for antibacterial action. Thus, the two polymers (PVA and PLA) through their characteristics optimize the mechanical properties and cell compatibility, presenting therapeutic benefits for medical purposes.

To overcome the limits related to the high hydrophobicity of PLA and the unsatisfactory cell adhesion of PVA, Datta et al. synthetized a new graft copolymer—phosphorylated polyvinyl alcohol-graft-polylactic acid (PPVA-g-LA). The synthesis reaction occurred in two steps: (1) esterification of the phosphorylated polyvinyl alcohol (PPVA) with lactic acid (LA), and (2) polymerization into PLA using stannous chloride as a catalyst. The obtained data showed an improvement in the hydrophilicity of the newly synthesized compound compared to both PPVA and PLA. Cell proliferation tests performed using MG-63 cells demonstrated that PPVA-g-LA can significantly improve cell proliferation and differentiation compared to the PPVA substrate and mitigates osteoblast function in comparison to PLA. Consequently, the synergy of these polymers results in enhanced cell proliferation and differentiation, surpassing the effects observed when PVA or PLA are used individually [310].

## 7. Conclusions and Future Perspectives

This review examined the performance of PVA-based membranes, emphasizing their significance in environmental and biomedical applications. The literature highlights PVA’s promising influence on membrane hydrophilicity, biocompatibility, and functionality, making it a key material in membrane technologies. Its versatility, tunability, and sustainability reinforce its important role in membrane development.

Continuous advancements in polymer engineering have led to the optimization of PVA-based membranes through techniques such as crosslinking, blending, and nanocomposite incorporation. These modifications enhance membrane properties, including selectivity, permeability, and long-term stability, thereby expanding their potential for high-performance applications in the biomedical, environmental, and industrial fields. Despite these advantages, tailoring the physicochemical properties of PVA membranes remains a significant challenge due to the limitations of the current synthesis methodologies. Although the modification techniques such as coating and grafting offer simple, cost-effective, and chemical-free solutions, concerns regarding long-term stability persist and justify further investigation.

A key shift in the research focuses on the transition from empirical membrane engineering toward advanced industrial applications, where fundamental thermodynamic and hydrodynamic modeling plays a critical role in optimizing membrane design. By integrating these theoretical approaches, researchers can systematically explore the experimental variables, enabling precise control over the membrane’s structure, surface properties, and mechanical integrity. Such advancements are essential for improving separation efficiency, enhancing antifouling properties, and ensuring chemical and mechanical resilience in practical applications.

Looking ahead, further efforts are needed to develop more efficient PVA-based membranes with enhanced multifunctionality and structural complexity. Achieving this goal will require the following: (i) exploration of a wider variety of PVA-based polymeric materials with tailored functionalities to meet specific application demands; (ii) improved control over synthesis steps to achieve precise morphological and structural characteristics; (iii) a systematic approach to experimental techniques and the valorization of the fundamental thermodynamic insights to improve design strategies and increase membrane performance. Addressing these challenges will stimulate innovation in membrane technology, encouraging the development of next-generation membranes for water purification, gas separation, pervaporation, and biomedical applications.

## Figures and Tables

**Table 1:** Empirical equations used in modeling transport mechanisms of membranes.

Law/ Author	Equation	Phenomenological Parameters/ Involved Parameters	Membrane Mechanism
Darcy [57,58]	$\bar{u}=-\frac{k}{\mu }\frac{dp}{dx}$	$\bar{u}$—average velocity	RO
		*k*—membrane permeability	
		*µ*—fluid viscosity	
Hagen–Poiseuille [57,58]	$\nu =\frac{\Delta p}{R}$	*ν*—flow rate	MF, UF, NF
		$R=\frac{8\eta L}{\pi r^{4}}$—viscous resistance	
		ε—surface porosity	
Carman–Kozeny [57]	$u=\frac{\varepsilon^{3}}{Kµ{\left(1-\varepsilon \right)}^{2}S_{0}}\frac{\Delta p}{L}$	*K*—Kozeny constant	MF, UF
		S_0_—specific surface of porous structure	
Nernst–Planck [59,60]	$J_{i}=\left(-D_{i\;}\frac{dc_{i}}{dx}\right)-\left(D_{i}z_{i}c_{i}\frac{F}{RT}\frac{d}{dx}\right)+\left(K_{i}c_{i}J_{v}\right)$	*Ji*—solute flux	NF, RO
		*Di*—diffusivity of solute *i*	
		*c_i_*—solute concentration at membrane surface	
		*x*—mole fraction of solute	
		*z_i_*—valence of solute	
		*F*—Faraday’s constant	
		*R*, *T*—gas constant and temperature	
		*Ψ*—electric potential	
		*K_i_*—distribution coefficient of solute	
		*J_v_*—volume flux	
Donnan [59,60]	$\Psi_{D}=\Psi_{m}-\Psi_{s}=c_{i}=C_{i}\cdot {\left(z_{i}\frac{F}{RT}\Delta \Psi_{D}\right)}^{n}$	*C_i_*—feed concentration of solute	NF, RO
		*Ψ_D_*—Donnan potential	
		*Ψ_m_*—electrical potential of solution	
		*Ψ_D_*—electrical potential of membrane	
Spiegler, Kedem, Katchalsky [61,62,63,64]	$\sigma =0.01397+0.00212\;{\cdot \;E}_{hyd}$	σ—membrane reflection coefficient	NF, RO, UF
		*E_hyd_*—hydration energy	
Geraldes [65]	$\frac{\delta_{\omega }}{h}{=15.5\;\left(\frac{l}{h}\right)}^{0.4}\;\cdot \;{Re}^{-0.4}{Sc}^{-0.63}{Re}_{p}^{-0.04}$ $\cdot \;\left(1-186{Sc}^{-0.1}{Re}_{p}^{-0.21}\right)$	*δ_ω_*—ionic concentration	NF
		*h*—boundary layer thickness	
		*l*—length of filter channel	
		*Re*, *Sc*—Reynolds and Schmidt numbers:	
		250 < *Re* < 1000	
		0.02 < *Rep* < 0.1	
		800 < *Sc* < 3200	
Shaalan [66]	$\frac{J}{J_{0}}=0.65exp\left(-0.13t\right)$ $exp\left(-0.031I\right)exp\left(0.896TOC\right)$ $\exp\left(1.23P\right)exp\left(-0.34d\right)$	*J*—solute flux	NF, UF
		*J_0_*—initial flux	
		*t*—time	
		*I*—ion strength	
		*TOC*—concentration	
		*P*—operating pressure	
		*d*—membrane cutoff	

**Table 2:** Overview of the biomedical applications of PVA membranes discussed in this review.

Applications	Key Characteristics	References
Drug delivery	Biocompatibility, controlled drug release, mucoadhesiveness, tunable mechanical properties, hydrophilic nature nanoparticle incorporation, pH-sensitive release	[218,221,228,229,230,231,232,233,234,235,236,237]
Wound dressing	High water absorption, oxygen permeability, antibacterial properties, mechanical strength, biodegradability	[238,239,240,241,242,243,244,245,246,247,248,249,250,251,252,253,254,255,256,257,258]
Tissue engineering and regenerative medicine	Cell adhesion, scaffold porosity, mechanical stability, biodegradability, tunable elasticity, bioactivity enhancement	[30,220,259,260,261,262,263,264,265,266,267,268,269,270,271,272,273,274]
Hemodialysis and artificial organs	Selective permeability, antifouling properties, hemocompatibility, mechanical durability, toxin adsorption	[83,275,276,277,278,279,280,281]
Other biomedical applications: periodontal treatment, ophthalmic, orthopedic, cancer therapy, immunotherapy, gene therapy, cosmetics	Adaptability, bioadhesion, sustained release, targeted therapy, soft tissue compatibility, non-toxicity	[282,283,284,285,286,287,288,289]

## Data Availability

Not applicable.
